# Health literacy, exercise self-efficacy, and physical activity in university students: a cross-sectional study

**DOI:** 10.3389/fpsyg.2026.1863857

**Published:** 2026-10-30

**Authors:** Ben Allen

**Affiliations:** Department of Psychology, University of Kansas, Lawrence, KS, United States

**Keywords:** eHealth literacy, health literacy, physical activity, self-efficacy, university students

## Abstract

**Background:**

Evidence of the association between health literacy and physical activity is inconsistent. Health literacy may support healthy behavior, but social cognitive theory suggests that acting on health information also depends on confidence to initiate and persist despite barriers. Exercise self-efficacy may therefore help explain when health literacy is associated with greater physical activity. We tested whether adding exercise self-efficacy improved models for weekly physical activity and estimated the cross-sectional indirect association between health literacy and physical activity via exercise self-efficacy.

**Methods:**

We conducted a cross-sectional study of 592 students at a large Midwestern public university in 2025 (median age = 18 years; 70.8% female). We assessed weekly physical activity using the International Physical Activity Questionnaire Short Form, alongside perceived digital and general health literacy, performance-based functional health literacy, and exercise self-efficacy. We compared a health literacy-centered model and a self-efficacy-extended model using structural equation modeling, 5,000-resample bootstrap confidence intervals, and repeated 10-fold cross-validation with five repeats using held-out predictive *R*^2^.

**Results:**

Adding exercise self-efficacy improved total-activity models across all three health literacy measures (bootstrapped Δ*R*^2^ = 0.112–0.128; mean pooled out-of-fold predictive Δ*R*^2^ = 0.110–0.127). Estimation of standardized indirect associations via exercise self-efficacy supported digital health literacy (*β* = 0.051, 95% CI = 0.017 to 0.090) and general health literacy (*β* = 0.067, 95% CI = 0.031 to 0.107), but not functional health literacy (*β* = 0.002, 95% CI = −0.029 to 0.032). Exploratory analyses by activity intensity provided evidence of associations for vigorous and moderate activity, whereas estimates for walking did not survive false discovery rate control. Sensitivity analyses using an alternate specification of total activity yielded the same qualitative pattern.

**Conclusion:**

Exercise self-efficacy improved models of physical activity across all three health literacy measures, while evidence supported indirect associations for perceived digital and general health literacy but not for performance-based functional health literacy. Exploratory analyses provided evidence of associations for moderate and vigorous activity, but the cross-sectional design precludes causal or temporal interpretation of the indirect associations.

## 1 Introduction

Physical activity is important for health, and young adulthood offers a substantial opportunity for campus-based health promotion (Kljajević et al., 2021; Lee et al., 2012; Huang et al., 2024). Health literacy can be foundational to health behavior because it shapes how individuals access, interpret, and incorporate health information into daily life (Nutbeam, 2008; Sørensen et al., 2012). However, health literacy is not a single construct. Perceived and performance-based measures of health literacy may reflect different underlying capacities, while digital health literacy concerns the ability to find, evaluate, and use health information from electronic resources (Moriishi et al., 2026; Norman and Skinner, 2006; Edwards et al., 2025).

University students provide an informative population for health behavior research because the transition to higher education increases personal responsibility while introducing academic, social, and scheduling demands that can disrupt established activity routines. For example, a recent systematic review found that time and environmental constraints, social influences, and goals or prioritization were among the most prominent determinants of university students’ physical activity, whereas knowledge was comparatively less prominent (Brown et al., 2024). This combination makes university students a useful population in which to examine whether associations between informational capabilities and physical activity are clearer when we also consider social-cognitive resources, such as confidence to act (Liu et al., 2024).

Health information alone may not be sufficient for sustained behavior change (Arlinghaus and Johnston, 2017; Hood et al., 2015; Matthews et al., 2024; Ginis et al., 2013). Social cognitive theory suggests that acting on such information also depends on self-efficacy, or confidence in one’s ability to act despite obstacles (Bandura, 1977). Moreover, exercise self-efficacy is consistently associated with physical activity (Young et al., 2014; Rovniak et al., 2002). Consistent with this, a prospective study of college students found temporally ordered associations linking digital and general health literacy to subsequent social-cognitive variables and, in turn, to later physical activity (Liu et al., 2024). These findings raise two related but distinct questions. The first is whether exercise self-efficacy explains and predicts physical activity beyond health literacy. The second is whether the cross-sectional pattern of relationships is consistent with an indirect association between health literacy and physical activity involving exercise self-efficacy. Answers to both questions may depend on how health literacy is measured. Perceived health literacy and exercise self-efficacy both involve self-appraisals of ability and may therefore be more closely related than performance-based health literacy and exercise self-efficacy (Moriishi et al., 2026; Norman and Skinner, 2006; Edwards et al., 2025). Accordingly, an observed indirect association involving perceived health literacy could reflect the hypothesized process, overlapping self-perceptions, or both.

Existing research has largely tested whether health literacy is associated with physical activity rather than whether exercise self-efficacy contributes explanatory and predictive information beyond health literacy (Buja et al., 2020; Lawrence et al., 2026). It remains unclear whether exercise self-efficacy adds information beyond health literacy, particularly across perceived and performance-based measures (Liu et al., 2024; McAnally and Hagger, 2023; Geboers et al., 2014). Previous studies have also often aggregated across activity intensities, potentially obscuring activity-specific patterns given that walking and more intensive physical activity can have partly different determinants and behavioral profiles (Craig et al., 2003; Rebar et al., 2016; Allen and Vella, 2015; Wendel-Vos et al., 2007). This possibility motivated exploratory analyses of vigorous, moderate, and walking activity rather than intensity-specific hypotheses.

Here, we compared health literacy-centered models for total physical activity with self-efficacy-extended models that also included exercise self-efficacy. These nested comparisons tested whether exercise self-efficacy provided incremental explained variance and out-of-sample predictive information beyond performance-based and perceived measures of health literacy. We separately estimated whether each health literacy measure had an indirect association with total physical activity involving exercise self-efficacy. We hypothesized that adding exercise self-efficacy would improve model performance and that each health literacy measure would show a nonzero indirect association with total physical activity involving exercise self-efficacy. Consistent with recommendations to emphasize indirect estimates rather than classifications of full versus partial mediation, our hypotheses concerned the indirect estimates themselves, not whether residual direct associations remained statistically supported (Hayes, 2022; Ledermann et al., 2025). The component-specific analyses were exploratory, and differences across health literacy measures and activity components were interpreted descriptively.

## 2 Materials and methods

### 2.1 Study design and participants

Participants were recruited through the Psychology Department SONA pool at a large Midwestern public university between September and December 2025. Eligible participants were university students aged 18 years or older. Data collection was conducted either in person in a campus laboratory or online via Qualtrics. In-person participants provided written electronic consent before completing anthropometric assessment, seated blood pressure measurement, and a computer-based questionnaire. Online participants reviewed an Institutional Review Board-approved information statement instead of signed documentation of consent and indicated consent by proceeding to the same questionnaire battery. Thus, questionnaire administration was electronic in both modes, although the surrounding testing context differed. Participation was voluntary. Participants could skip any question or discontinue at any time without penalty. Class research credits were awarded for participation. Total participation time was approximately 60 min or less. The University of Kansas Institutional Review Board approved the study protocol and consent procedures (#00152012) on August 13, 2025. The sample size was determined by the number of eligible students who participated during the September–December 2025 recruitment period. No formal *a priori* sample-size calculation was used.

A total of 642 responses were originally recorded (395 online; 247 in person). For the present analysis, 50 records were excluded because they comprised blank or demographic-only entries, yielding an analytic base sample of 592 students (347 online; 245 in person). Table 1 shows descriptive statistics for the analytic base sample. Self-reported age and sex information was available for 589 students. Protocol-based IPAQ processing yielded valid summary outcomes for 560 participants, while general health literacy and exercise self-efficacy had additional missingness. Consequently, complete-case regression samples were 554 for models using digital and functional health literacy and 541 for the model using general health literacy.

**Table 1:** Sample characteristics and descriptive statistics.

Sample characteristic	Descriptive statistic
Overall *N*	592
Age (years), median [IQR]	18 [18, 19]; *n* = 589
Sex, female	417/589 (70.8%)
Sex, male	172/589 (29.2%)
Physical activity	
IPAQ total MET-min/week, median [IQR]	4158.00 [2251.88, 6297.75]; *n* = 560
IPAQ vigorous MET-min/week, median [IQR]	1280.00 [240.00, 2880.00]; *n* = 560
IPAQ moderate MET-min/week, median [IQR]	480.00 [240.00, 1200.00]; *n* = 560
IPAQ walking MET-min/week, median [IQR]	1386.00 [792.00, 2772.00]; *n* = 560
IPAQ sitting min/weekday, median [IQR]	300 [240, 420]; *n* = 583
Physical activity category	
Low	23/560 (4.1%)
Moderate	149/560 (26.6%)
High	388/560 (69.3%)
Health literacy and self-efficacy	
Exercise self-efficacy total score, median [IQR]	56 [44, 72]; *n* = 591
eHEALS (digital health literacy), median [IQR]	32.00 [28.00, 33.25]; *n* = 592
HLS-EU-Q16 / GHL index, median [IQR]	34.38 [31.25, 39.58]; *n* = 576
Newest Vital Sign (performance-based functional HL), median [IQR]	5 [3, 6]; *n* = 592

Values are *n*/*N* (%) or median [IQR]. Variable-specific *n* values are shown when missingness was present. IPAQ activity summaries and categories use *n* = 560 because 32 participants lacked valid summary outcomes after protocol-based processing; the analytic base remains *N* = 592. IPAQ, International Physical Activity Questionnaire; eHEALS, eHealth Literacy Scale; HLS-EU-Q16, European Health Literacy Survey Questionnaire 16-item short form; GHL, general health literacy; IQR, interquartile range.

### 2.2 Measures

#### 2.2.1 Physical activity

The primary outcome was total metabolic equivalent minutes per week (MET-min/week) measured using the International Physical Activity Questionnaire Short Form (IPAQ-SF) (Craig et al., 2003). Primary models used raw weekly total MET-min/week to preserve interpretability. Because the total score remained strongly right-skewed, all adjusted total-activity models were repeated using log(1 + total MET-min/week) as a sensitivity analysis. To clarify contributors to the total IPAQ score, we performed a secondary decomposition of total MET-min/week into vigorous, moderate, and walking MET-min/week. Because these component outcomes were also right-skewed, the component models used the same log transformation.

IPAQ-SF responses were processed as follows. For each activity type, daily duration was calculated as (60 × hours) + minutes, with a blank component treated as zero. As a unit-entry correction, an hours-field value of exactly 15, 30, 45, 60, or 90 was interpreted as minutes rather than hours. These values correspond to common minute increments but would represent 15–90 h of a single activity per day if interpreted literally. This correction affected 10 fields across nine participant records: one vigorous, five moderate, and four walking fields. Eight participants had one corrected field, and one had two. In all 10 instances, the corresponding minutes field was blank or zero. No corrected entry required combining a reinterpreted hours-field value with additional positive minutes (Supplementary Table S22).

We did not assign IPAQ summary scores to participants who reported positive activity days but were missing both duration fields. We also excluded participants from summary scoring when total pre-truncation duration across vigorous, moderate, and walking activity exceeded 960 min/day. Bouts shorter than 10 min/day and their associated activity days were recoded to zero. Remaining durations were truncated at 180 min/day within each activity type. MET-min/week was then calculated using values of 8.0, 4.0, and 3.3 METs for vigorous, moderate, and walking activity, respectively. Overall, 32 of 592 participants lacked valid IPAQ summary scores: 12 exceeded 960 min/day and 20 reported positive activity days without sufficient duration information. Thus, 560 participants had valid summary scores (see Supplementary Table S22).

For descriptive purposes, activity categories were computed using the November 2005 International Physical Activity Questionnaire scoring protocol. High activity required either at least 3 days of vigorous-intensity activity and at least 1,500 total physical activity MET-min/week, or at least 7 days of any combination of walking, moderate-intensity, and vigorous-intensity activity and at least 3,000 total physical-activity MET-min/week. Moderate activity required not meeting the high criteria and meeting at least one of the following: at least 3 days of vigorous-intensity activity for at least 20 min/day; at least 5 days of moderate-intensity activity and/or walking for at least 30 min/day; or at least 5 days of any combination of walking, moderate-intensity, and vigorous-intensity activity and at least 600 total physical-activity MET-min/week. All other participants were classified as low activity.

#### 2.2.2 Exercise self-efficacy

Exercise self-efficacy was the intervening variable in the statistical indirect-association models. The nine-item Self-Efficacy for Exercise scale asks respondents to rate confidence in exercising despite common barriers on a 0–10 response scale, yielding a 0 to 90 total score, with higher scores corresponding to greater exercise self-efficacy (Resnick and Jenkins, 2000). The total score was recomputed directly from item-level responses and prorated as the completed-item mean multiplied by nine when at least seven of nine items were present. If fewer than seven items were present, the score was set to missing. Internal consistency in the present sample was high among participants with complete item sets (Cronbach’s *α* = 0.93, *n* = 588; Supplementary Table S13).

#### 2.2.3 Health literacy

Health literacy was operationalized using three distinct measures. Digital health literacy was assessed with the 8-item electronic Health Literacy Scale (eHEALS), whose items are rated from 1 (strongly disagree) to 5 (strongly agree), yielding a total score of 8–40. Higher scores indicated a greater perceived ability to find, evaluate, and use online health information (Norman and Skinner, 2006). Complete eHEALS responses were summed. The completed-item mean was prorated to the eight-item total metric for participants with only seven answered items (*n* = 4).

General health literacy was assessed with the 16-item European Health Literacy Survey Questionnaire short form (HLS-EU-Q16). Items were coded from 1 to 4. When at least 14 of 16 items were answered, we calculated the mean response across the available items and applied the published HLS-EU general-index transformation, (mean item response − 1) × (50/3), yielding a 0 to 50 score, where higher values show greater perceived general health literacy (Pelikan and Ganahl, 2017; Lorini et al., 2019). We selected this continuous scoring approach to retain the ordinal response information rather than dichotomizing responses into the conventional 0–16 HLS-EU-Q16 score.

Performance-based functional health literacy was measured with the Newest Vital Sign, a six-item nutrition-label interpretation and numeracy assessment scored from 0 to 6, with higher scores showing greater functional health literacy (Weiss et al., 2005). All three health literacy scores were analyzed as continuous variables. Internal consistency based on complete item sets showed a Cronbach’s *α* = 0.92 for eHEALS (*n* = 588), *α* = 0.89 for HLS-EU-Q16 (*n* = 503), and *α* = 0.75 for the Newest Vital Sign (*n* = 592; Supplementary Table S13).

### 2.3 Statistical analysis

Analyses were conducted in R 4.3.3 using lavaan 0.6–19, manymome 0.2.9, mice 3.16.0, rsample 1.2.1, and yardstick 1.3.1. We compared a health literacy-centered model containing health literacy, age, and sex with a self-efficacy-extended model that additionally contained exercise self-efficacy. These labels are shorthand for the statistical models. Age and sex were selected *a priori* as basic demographic covariates potentially associated with the outcome and predictors, rather than on the basis of their observed statistical significance. Because the design was cross-sectional, product-of-coefficients estimates are described as indirect associations rather than causal mediation. Missing data were handled according to the analysis: SEMs used full-information maximum likelihood, nested regression comparisons used complete cases to preserve paired samples, and predictive cross-validation used multiple imputation within training folds.

For each health literacy measure, we compared the health literacy-centered and self-efficacy-extended models using complete-case linear regressions. Nonparametric bootstrapping with 5,000 resamples provided 95% confidence intervals for the change in explained variance (Δ*R*^2^) and standardized regression coefficients. Complementary reverse-order analyses evaluated the addition of health literacy to a model containing exercise self-efficacy, age, and sex. Adjusted and unadjusted comparisons are reported in Supplementary Tables S5, S9, S10.

Regression diagnostics were assessed in the paired complete-case samples. Potentially influential observations and leverage were examined using hat values, Cook’s distances, and externally studentized residuals. Heteroscedasticity was assessed using Koenker–Breusch–Pagan tests, and functional form using Ramsey RESET tests with squared and cubed fitted values. Screening thresholds and model-specific results are reported in Supplementary Table S23.

SEMs estimated the cross-sectional indirect association of health literacy with physical activity through exercise self-efficacy. Health literacy, age, and sex predicted exercise self-efficacy. Health literacy, exercise self-efficacy, age, and sex predicted physical activity. The indirect and total associations were defined as *ab* and *c′ + ab*, respectively. Models were estimated in lavaan using full-information maximum likelihood, random exogenous variables (fixed.*x* = FALSE), and 5,000 bootstrap resamples. All 592 participants contributed available data to each primary SEM. Estimation options and observation counts are reported in Supplementary Table S16. Exact model syntax and complete analysis code are available on the Open Science Framework (see Data Availability Statement). Each observed-variable path model was just identified (df = 0), so global fit indices were not interpreted. For numerical stability, total MET-min/week was divided by 1,000 during estimation, and unstandardized estimates were subsequently returned to their original units.

Predictive performance was evaluated using five repeats of 10-fold cross-validation. The forward and reverse comparisons were conducted separately and therefore used separately generated resampling partitions and imputations. Within each comparison, the nested models used identical partitions, completed datasets, and evaluable assessment observations.

Within each fold, training and assessment rows were supplied together to the MICE algorithm, but imputation models were estimated only from the training observations and then applied to incomplete predictors in both sets. Five completed datasets were generated, each after five iterations of chained equations. Predictive mean matching was used for age, exercise self-efficacy, and general health literacy, and logistic regression was used for sex. Each incomplete predictor was imputed from the other model predictors, with self-prediction blocked. Digital and functional health literacy required no imputation, and physical activity was neither included in the imputation equations nor imputed. Models were fitted separately to the five completed training datasets, and predictions for each assessment participant were averaged across imputations before calculating *R*^2^ and RMSE.

All 592 participants were assigned to resampling partitions. The 32 participants without valid IPAQ outcomes could contribute observed predictor information to training-fold imputation but not to outcome-model fitting or performance evaluation. Assessment folds therefore contained 51–60 evaluable outcomes, and each repeat yielded pooled out-of-fold predictions for 560 participants. Predictive *R*^2^ was calculated from the pooled predictions as 1 − residual sum of squares divided by total sum of squares. Predictive Δ*R*^2^ was the difference between the model after and before adding the focal predictor. Reported values are arithmetic means of the five repeat-specific estimates.

Exploratory component analyses otherwise followed the primary modeling and cross-validation procedures. Comparisons across activity components and health literacy measures were descriptive because direct contrasts were not conducted. Primary total-activity indirect associations were evaluated using 95% percentile-bootstrap confidence intervals and were not multiplicity-adjusted. For the component analyses, Benjamini–Hochberg adjustment was applied separately within vigorous activity, moderate activity, and walking (Benjamini and Hochberg, 1995). Each family contained the three indirect-association hypotheses for digital, general, and functional health literacy. Inputs were lavaan’s two-sided normal-reference *p*-values for *ab*, calculated using bootstrap standard errors. Because the confidence intervals were obtained separately from percentiles of the empirical bootstrap distribution, they were not exact inverses of these tests. Family assignments, unadjusted *p*-values, confidence intervals, and adjusted *q*-values are reported in Supplementary Table S19.

Collection-mode groups were compared using Mann–Whitney U tests for continuous variables and a chi-square test for categorical variables (Supplementary Table S14). All three primary SEMs were also refitted with collection mode added to both structural equations (Supplementary Table S15). Additional sensitivity analyses omitted age and sex, used complete-case SEMs, modeled log(1 + total MET-min/week), restricted the general health literacy SEM to complete HLS-EU-Q16 item sets, and excluded the nine records affected by the IPAQ unit-entry correction (Supplementary Tables S8-S10, S18, S21, S22). Participants with and without valid IPAQ outcomes were compared descriptively using distributional summaries and standardized mean differences (Supplementary Table S17).

## 3 Results

### 3.1 Sample characteristics and activity distribution

The analytic base sample comprised 592 students, with a median age of 18 years. Among the 589 students with sex data, 417 (70.8%) were female and 172 (29.2%) were male (Table 1). Descriptive distributions by collection mode are reported in Supplementary Table S14. Median general health literacy was slightly higher for the in-person group (35.42 vs. 33.33), and the largest observed mode difference involved the Newest Vital Sign (median 5 vs. 4). Overall, 168 of 592 participants (28.4%) achieved the maximum NVS score of 6, and 308 (52.0%) scored 5 or 6 (Supplementary Table S20). After processing the IPAQ responses, 560 participants had valid activity summary scores, with a median total of 4,158.00 MET-min/week (IQR, 2,251.88-6,297.75). Among these participants, 23/560 (4.1%) were classified as Low, 149/560 (26.6%) as Moderate, and 388/560 (69.3%) as High.

### 3.2 Forward and reverse comparisons showed asymmetric incremental prediction

In the complete-case analyses, the self-efficacy-extended models explained 0.154–0.159 of the variance in total weekly physical activity (Figure 1; Table 2). Adding exercise self-efficacy increased in-sample *R*^2^ by 0.120 (95% CI, 0.066 to 0.186) for digital health literacy, 0.112 (95% CI, 0.058 to 0.178) for general health literacy, and 0.128 (95% CI, 0.069 to 0.193) for functional health literacy. In repeated cross-validation, mean pooled out-of-fold *R*^2^ increased from 0.012 to 0.134 (Δ*R*^2^ = 0.122), from 0.022 to 0.133 (Δ*R*^2^ = 0.110), and from 0.007 to 0.134 (Δ*R*^2^ = 0.127), respectively. Thus, adding exercise self-efficacy improved held-out prediction relative to the health literacy-centered models, although the absolute predictive performance of the self-efficacy-extended models remained modest at approximately 13% of held-out variation in total physical activity.

**Figure 1:**
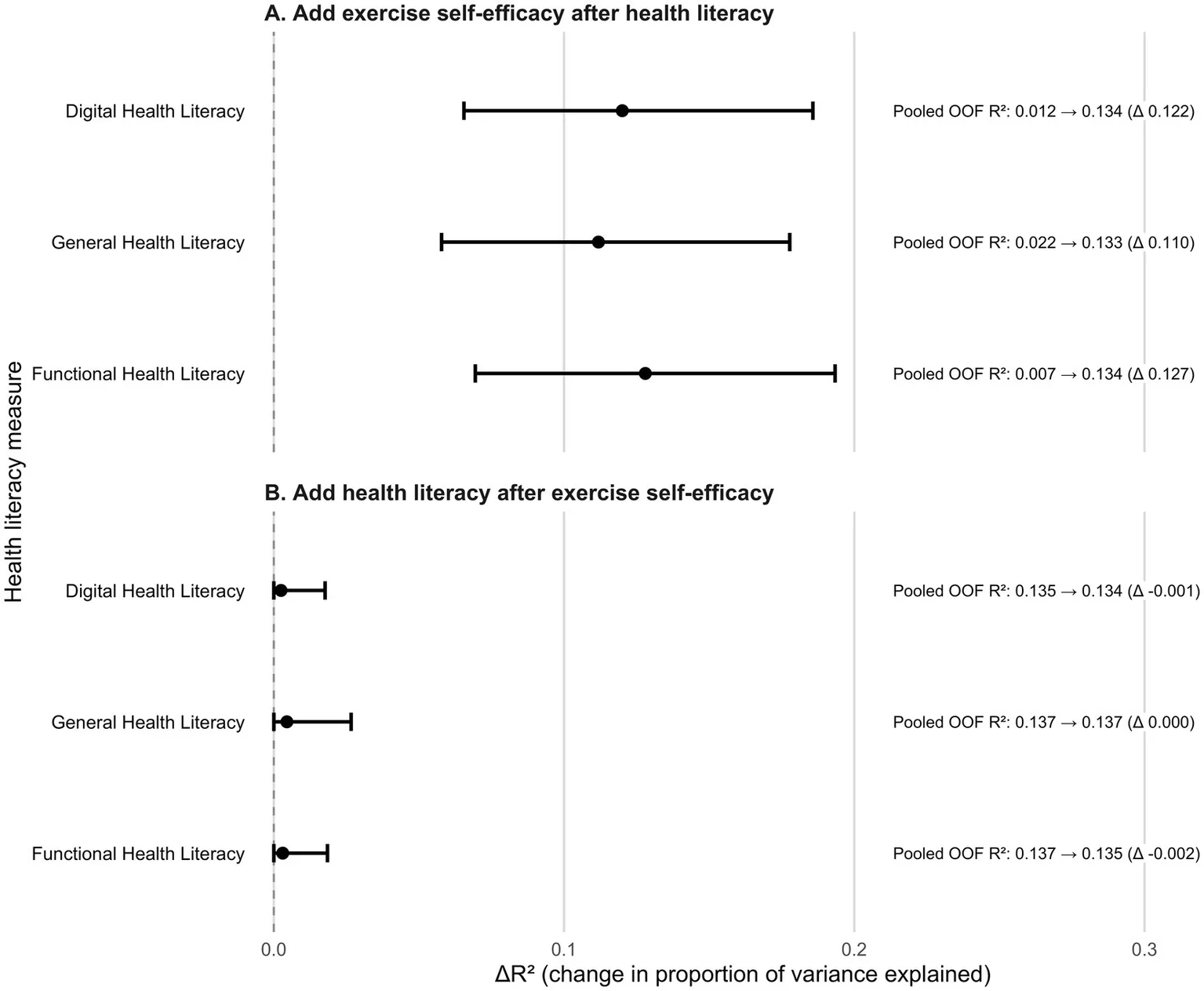
Forward and reverse incremental model comparisons. Points and horizontal bars show bootstrapped in-sample Δ*R*^2^ estimates and 95% confidence intervals for adding **(A)** exercise self-efficacy to health literacy, age, and sex and **(B)** health literacy to exercise self-efficacy, age, and sex. Annotations show mean pooled out-of-fold predictive *R*^2^ before and after adding the focal predictor and the corresponding Δ*R*^2^ across five repeats. Forward and reverse comparisons used separately generated resampling partitions and imputations. Nested models within each comparison shared both. Full-model pooled OOF *R*^2^ may differ slightly between panels despite containing the same predictors. OOF, out-of-fold; SEE, exercise self-efficacy.

**Table 2:** Global model comparison and indirect-association results for weekly physical activity.

Health literacy model	*a* path	*b* path	*c*′ path	Indirect and total associations	Physical activity *R*^2^	Δ*R*^2^ after adding self-efficacy
Digital health literacy	*B* = 0.482 [0.174, 0.785] *β* = 0.138	*B* = 58.462 [43.366, 73.318] *β* = 0.367	*B* = 27.066 [−21.164, 74.887] *β* = 0.049	Indirect *β* = 0.051 [0.017, 0.090] Total *B* = 55.272 [4.391, 104.019] *β* = 0.099	0.154	0.120 [0.066, 0.186]
General health literacy	*B* = 0.527 [0.266, 0.773] *β* = 0.185	*B* = 57.361 [41.822, 72.300] *β* = 0.360	*B* = 28.989 [−19.384, 77.985] *β* = 0.064	Indirect β = 0.067 [0.031, 0.107] Total *B* = 59.192 [11.859, 107.065] *β* = 0.130	0.159	0.112 [0.058, 0.178]
Functional health literacy	*B* = 0.054 [−0.840, 0.918] *β* = 0.005	*B* = 59.658 [45.034, 74.041] *β* = 0.374	*B* = −92.227 [−237.538, 49.498] *β* = −0.053	Indirect *β* = 0.002 [−0.029, 0.032] Total *B* = −89.020 [−239.508, 60.337] *β* = −0.052	0.155	0.128 [0.069, 0.193]

Complete-case regression n was 554 for the digital and functional health literacy models and 541 for the general health literacy model. Structural equation models used full-information maximum likelihood with missing = “fiml,” fixed.*x* = FALSE, and meanstructure = TRUE; lavaan:lavInspect(fit, “nobs”) returned 592 for each primary SEM. Each inferential bootstrap procedure requested 5,000 resamples; primary and component SEMs retained at least 99.5% valid bootstrap refits. Indirect associations are standardized estimates with 95% bootstrap confidence intervals. Total associations are SEM total effects (*c*′ + *ab*), presented as unstandardized *B* with 95% bootstrap confidence intervals and standardized *β*. Physical activity *R*^2^ refers to the complete-case regression full model.

Complementary reverse-order comparisons evaluated the information contributed by health literacy after the model included exercise self-efficacy, age, and sex. Adding digital, general, and functional health literacy increased in-sample *R*^2^ by 0.0025 (95% CI, 0.0000 to 0.0177), 0.0045 (95% CI, 0.0000 to 0.0266), and 0.0031 (95% CI, 0.0000 to 0.0185), respectively (Figure 1). The corresponding mean pooled out-of-fold changes were −0.0006, 0.0003, and −0.0023. Exercise self-efficacy therefore improved held-out prediction after the model included health literacy, whereas health literacy did not improve held-out prediction after the model included exercise self-efficacy.

Diagnostic screening to flag potentially influential observations showed that each model contained observations exceeding the prespecified screening thresholds for leverage, Cook’s distance, and absolute externally studentized residuals (Supplementary Table S23). However, no Cook’s distance exceeded 1, and no observations were excluded. Koenker–Breusch–Pagan tests indicated heteroscedasticity in both the health literacy-centered and self-efficacy-extended models involving general health literacy (*p* = 0.014 and 0.028, respectively), but not in the models involving digital or functional health literacy. Ramsey RESET tests suggested that the additive linear specification might not fully capture the conditional mean in the three self-efficacy-extended models (*p* = 0.009, 0.005, and 0.033). Because RESET is an omnibus test, these findings do not identify a particular nonlinear relationship or interaction. Given these diagnostic findings, we interpreted the raw-scale results alongside the log-transformed sensitivity analysis. The forward-comparison pattern was unchanged when total activity was modeled as log(1 + total MET-min/week) (Supplementary Table S8).

### 3.3 Indirect associations via self-efficacy supported for perceived health literacy

Cross-sectional SEMs supported standardized indirect associations through exercise self-efficacy for digital health literacy (*β* = 0.051, 95% CI 0.017 to 0.090) and general health literacy (*β* = 0.067, 95% CI 0.031 to 0.107), but not for functional health literacy (*β* = 0.002, 95% CI −0.029 to 0.032; Figure 2; Table 2). For digital and general health literacy, higher literacy was associated with higher exercise self-efficacy, and higher exercise self-efficacy was associated with greater weekly physical activity.

**Figure 2:**
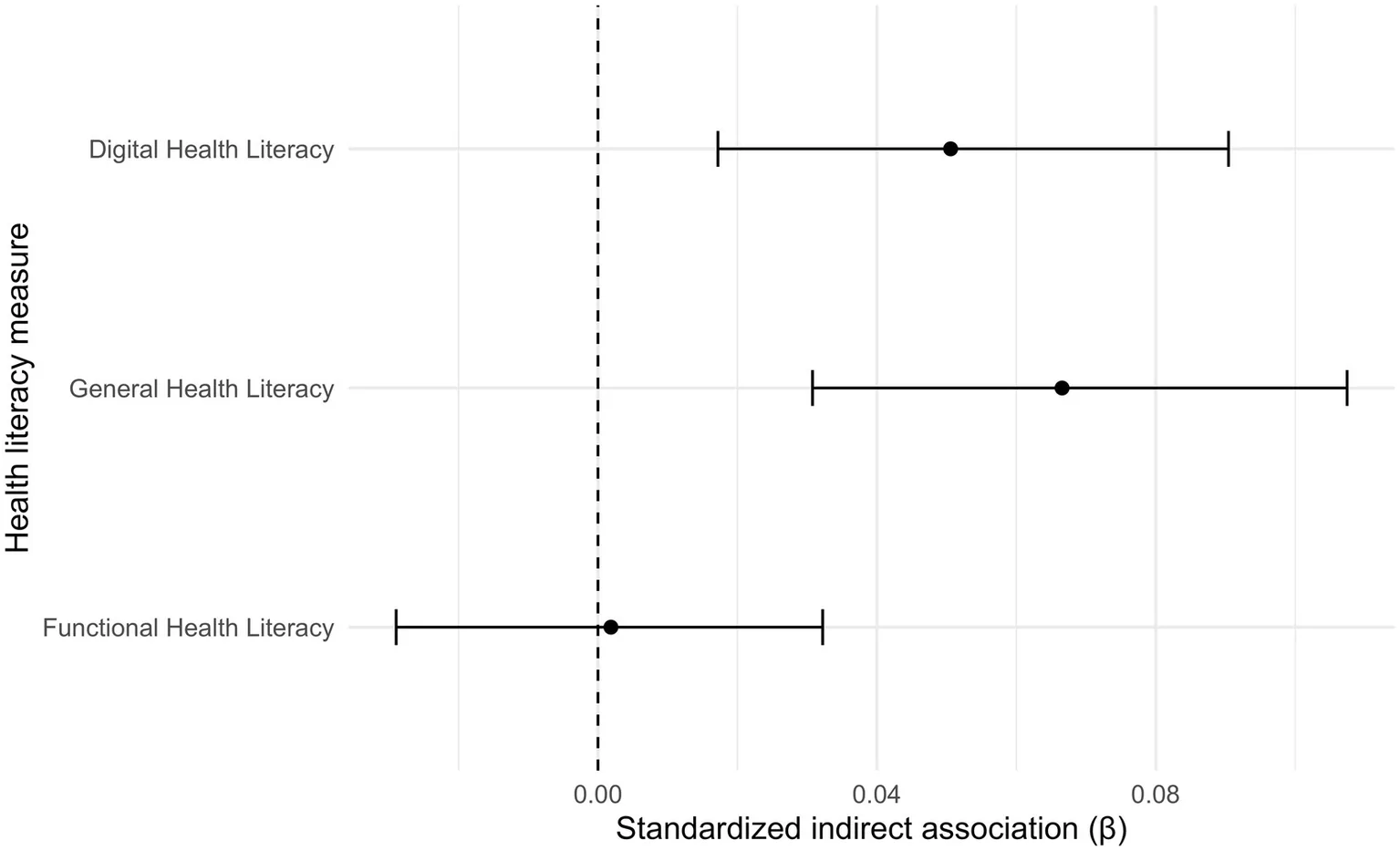
Standardized cross-sectional indirect associations of health literacy with total weekly physical activity through exercise self-efficacy. Points are estimates and horizontal bars are 95% bootstrap confidence intervals.

After collection mode was added to both structural equations, the standardized indirect associations were 0.050 (95% CI 0.019 to 0.089) for digital health literacy, 0.067 (95% CI 0.030 to 0.109) for general health literacy, and 0.004 (95% CI −0.028 to 0.036) for functional health literacy (Supplementary Table S15). These estimates remained similar in magnitude and precision to the primary estimates, and the substantive interpretation was unchanged. The overall indirect-association pattern was also unchanged in the log-transformed sensitivity analyses (Supplementary Table S8).

### 3.4 Activity-specific estimates showed a descriptive ordering

The component analyses showed a consistent descriptive ordering, although we did not formally contrast differences among components (Table 3; Figure 3). For digital and general health literacy, the small-to-modest standardized indirect associations were largest for vigorous activity (*β* = 0.057–0.077), smaller for moderate activity (*β* = 0.030–0.043), and smallest for walking (*β* = 0.015–0.019). Vigorous and moderate associations survived Benjamini–Hochberg false discovery rate control, whereas the walking associations did not (both *q* = 0.104). Functional health-literacy indirect associations were unsupported across all three components. The self-efficacy-extended models achieved mean pooled out-of-fold *R*^2^ values of 0.191–0.196 for vigorous activity, 0.039–0.041 for moderate activity, and 0.019–0.024 for walking. The corresponding predictive Δ*R*^2^ values were 0.153–0.163, 0.039–0.045, and 0.007–0.009.

**Table 3:** Physical activity component models.

Health literacy model	Physical activity component	Standardized indirect association *β* [95% CI]	In-sample SEE Δ*R*^2^ [95% CI]	Pooled OOF predictive Δ*R*^2^
Digital health literacy	Vigorous	0.057 [0.021, 0.093]^a^	0.152 [0.100, 0.206]	0.1559
Digital health literacy	Moderate	0.030 [0.010, 0.056]^a^	0.040 [0.013, 0.079]	0.0391
Digital health literacy	Walking	0.015 [0.002, 0.032]	0.013 [0.001, 0.033]	0.0088
General health literacy	Vigorous	0.077 [0.035, 0.120]^a^	0.146 [0.095, 0.201]	0.1530
General health literacy	Moderate	0.043 [0.018, 0.074]^a^	0.047 [0.016, 0.092]	0.0447
General health literacy	Walking	0.019 [0.002, 0.042]	0.013 [0.001, 0.033]	0.0080
Functional health literacy	Vigorous	0.002 [−0.033, 0.037]	0.161 [0.108, 0.219]	0.1632
Functional health literacy	Moderate	0.001 [−0.017, 0.020]	0.043 [0.015, 0.084]	0.0403
Functional health literacy	Walking	0.001 [−0.008, 0.011]	0.012 [0.001, 0.032]	0.0071

Each component outcome was modeled as log(1 + component MET-min/week). Complete-case regression *n* was 554 for the digital and functional health literacy models and 541 for the general health literacy model.

a Indirect association survived false discovery rate control using the Benjamini–Hochberg procedure.

Δ*R*^2^ values are in-sample nested-model increments with percentile bootstrap uncertainty intervals; because nested-model Δ*R*^2^ is bounded at zero, these intervals are not treated as conventional null-hypothesis tests. Each inferential bootstrap procedure requested 5,000 resamples; component SEMs retained at least 99.5% valid bootstrap refits. The pooled OOF predictive Δ*R*^2^ column is the mean across five repeats of the difference in traditional predictive *R*^2^ (1 − SSE/SST), calculated after pooling all out-of-fold predictions within each repeat. OOF, out-of-fold.

**Figure 3:**
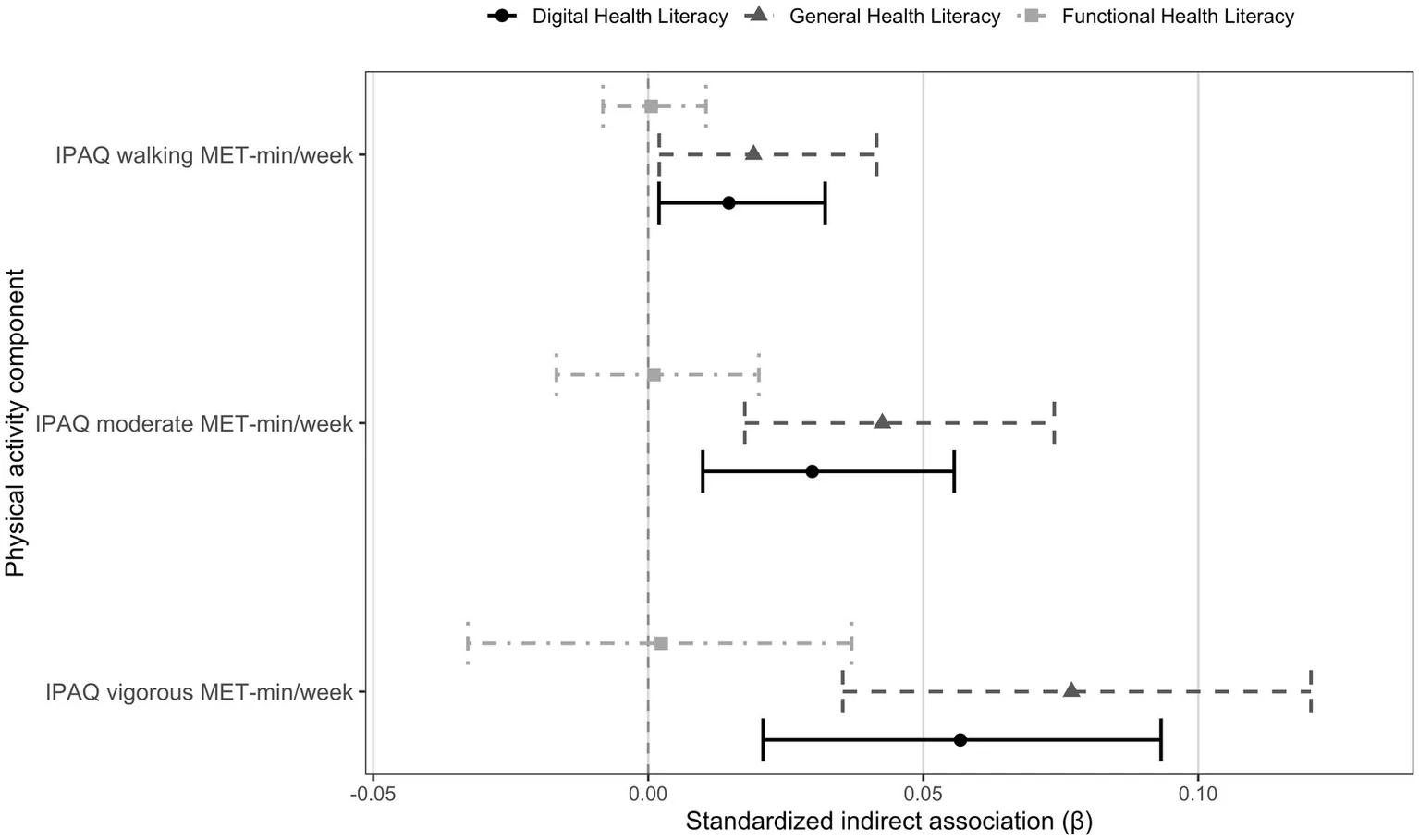
Standardized cross-sectional indirect associations for vigorous, moderate, and walking activity across the three health-literacy measures. Each physical-activity component was modeled as log(1 + MET-min/week). Points and horizontal bars show standardized estimates and 95% percentile-bootstrap confidence intervals. These unadjusted confidence intervals and the Benjamini–Hochberg-adjusted decisions reported in Table 3, Supplementary Table S19 are distinct inferential summaries and need not yield identical conclusions. The ordering across activity components is descriptive; component differences were not formally contrasted.

### 3.5 Missing-data and outcome-availability sensitivities

Among the 576 participants with an analyzable HLS-EU-Q16 score, 22 completed 14 items, 51 completed 15 items, and 503 completed all 16 items. 16 additional participants completed fewer than 14 items and were not scored (Supplementary Table S21). Restricting the general health literacy SEM to the 503 complete-item respondents yielded a standardized indirect association of *β* = 0.063 (95% CI, 0.026 to 0.108), compared with *β* = 0.067 (95% CI, 0.031 to 0.107) in the primary analysis. The substantive interpretation was unchanged.

After excluding the nine participants with corrected IPAQ hour fields, the standardized indirect associations were 0.051 (95% CI, 0.019 to 0.089) for digital health literacy, 0.067 (95% CI, 0.029 to 0.109) for general health literacy, and 0.003 (95% CI, −0.028 to 0.033) for functional health literacy. These estimates were similar to the primary estimates, and the substantive interpretation was unchanged (Supplementary Table S22).

To assess whether the primary full-information maximum likelihood results depended on the missing-data approach, we estimated the three SEMs using only participants with complete data on all model variables. Standardized indirect associations remained supported for digital health literacy (*β* = 0.048, 95% CI 0.016 to 0.085; *n* = 554) and general health literacy (*β* = 0.082, 95% CI 0.042 to 0.126; *n* = 541), but not for functional health literacy (*β* = 0.007, 95% CI −0.024 to 0.038; *n* = 554). Thus, the complete-case analyses reproduced the primary indirect-association pattern (Supplementary Table S18).

Among the 592 participants, 560 had a valid IPAQ summary outcome, 12 had an unavailable outcome because pre-truncation duration exceeded 960 min/day, and 20 had an unavailable outcome because positive activity days lacked sufficient duration information. Participants with and without valid IPAQ summary outcomes were descriptively similar in age, sex, exercise self-efficacy, digital health literacy, and general health literacy (absolute standardized mean differences ≤0.20 after rounding). The largest difference was for the Newest Vital Sign: the median was 5 among participants with valid IPAQ outcomes and 3 among those with unavailable outcomes (standardized mean difference = 0.90). The proportion assessed online was 58.0 and 68.8%, respectively (standardized mean difference = −0.22; Supplementary Table S17).

## 4 Discussion

In this study, exercise self-efficacy contributed substantial unique information beyond health literacy to models for weekly physical activity. Across three different measures of health literacy, adding exercise self-efficacy increased explained variance and improved held-out prediction. Conversely, adding health literacy did not improve held-out prediction after the model included exercise self-efficacy. Although the self-efficacy-extended models outperformed the health literacy-centered models, their absolute predictive performance remained modest. Digital and general health literacy showed cross-sectional indirect associations with total physical activity involving exercise self-efficacy, whereas functional health literacy did not. In exploratory component analyses, the corresponding indirect associations for digital and general health literacy were supported for vigorous and moderate activity, whereas the walking associations did not survive false discovery rate control. Functional health literacy showed no supported indirect associations across activity components.

### 4.1 Incremental contribution of exercise self-efficacy

The complementary model comparisons showed an asymmetric pattern. Adding exercise self-efficacy to models of total physical activity that included health literacy improved both explained variance and held-out prediction. In contrast, adding health literacy to models that already included exercise self-efficacy did not improve held-out prediction. That exercise self-efficacy contributed incremental explanatory and predictive information is consistent with social cognitive theory’s emphasis on perceived capability in acting despite barriers (Bandura, 1977; Young et al., 2014). In exploratory activity-specific analyses, the incremental estimates were numerically larger for vigorous and moderate activity than for walking, though we did not formally test contrasts between the activity components. Despite these incremental gains, the absolute predictive performance of the self-efficacy-extended models remained modest. Thus, exercise self-efficacy was statistically informative within health literacy-centered models, but predicting physical activity will require further information besides health literacy and self-efficacy.

### 4.2 Indirect associations and exploratory activity-specific patterns

In the primary total-activity analyses, perceived digital and general health literacy showed cross-sectional indirect associations with physical activity involving exercise self-efficacy, whereas performance-based functional health literacy did not. For the perceived health literacy measures, this pattern is consistent with the hypothesized sequence linking health literacy, exercise self-efficacy, and physical activity and with prospective evidence for temporal ordering among related constructs (Liu et al., 2024). However, the estimates in the present study are cross-sectional and cannot establish a sequence or causal mediation.

The activity-specific analyses provided an exploratory extension of the total-activity findings. For perceived digital and general health literacy, indirect associations involving exercise self-efficacy met the false-discovery-rate-adjusted threshold for vigorous and moderate activity, whereas the corresponding walking associations did not. Our findings did not support any component-specific indirect association for functional health literacy. One explanation for this pattern is that exercise self-efficacy is more closely aligned with activities requiring deliberate effort and persistence despite barriers. Walking may depend more strongly on routine, environmental context, transportation, or necessity (Rebar et al., 2016; Horacek et al., 2018). Thus, although the exploratory analyses showed a descriptive pattern across activity components, future studies should directly contrast the components to test whether the indirect associations differ by activity intensity.

### 4.3 Health literacy and the pattern of indirect association

While we found evidence of indirect associations for perceived digital and general health literacy but not for performance-based functional health literacy, our health literacy measures differed simultaneously in response format, substantive content, breadth, and score distribution. For example, the eHEALS assesses perceived competence in finding, evaluating, and using electronic health information (Norman and Skinner, 2006), whereas the HLS-EU-Q16 assesses perceived difficulty in processing health information more broadly (Pelikan and Ganahl, 2017; Lorini et al., 2019). The Newest Vital Sign instead assesses performance on a specific nutrition-label literacy and numeracy task (Moriishi et al., 2026; Weiss et al., 2005). As a result, we cannot uniquely attribute the observed pattern to self-appraisal versus performance-based measurement (Moriishi et al., 2026; Norman and Skinner, 2006; Edwards et al., 2025).

Comparison with previous research is complicated by differences in health literacy measurement. For example, Liu et al. (2024) used a brief general health literacy screener focused on hospital materials and forms and found no association between perceived general health literacy and self-efficacy for physical activity. In contrast, our study did show evidence of a cross-sectional indirect association with physical activity involving exercise self-efficacy, but our measure of general health literacy (HLS-EU-Q16) was broader and included health promotion content. Future studies are needed to test whether this discrepancy reflects instrument content or other differences between constructs or studies.

There are additional measurement factors that may explain the differing patterns observed across the health literacy measures. Perceived health literacy and exercise self-efficacy both rely on self-appraisal. Their association may therefore partly reflect shared method variance or overlap between the abilities participants were asked to appraise. Moreover, the Newest Vital Sign assesses a relatively narrow range of skills, and its scores were concentrated at the upper end of the distribution. These features may have limited its ability to distinguish among participants with higher functional health literacy. Future studies should directly compare perceived and performance-based health-literacy measures that assess more closely aligned content and provide adequate variability throughout the score range.

### 4.4 Strengths and limitations

A principal strength of this study is the complementary analyses that separated important research questions. Our nested design allowed the assessment of whether exercise self-efficacy adds information beyond health literacy, how well models with self-efficacy predict held-out observations of physical activity, and whether health literacy is indirectly associated with physical activity through exercise self-efficacy. Moreover, sensitivity analyses reproduced the substantive pattern, including when total activity was log-transformed. However, while this consistency is supportive, the diagnostic findings warrant caution in interpreting the raw-scale linear-model estimates.

A key limitation is the cross-sectional design, which cannot establish the temporal order or causal mediation of health literacy, self-efficacy, and physical activity (Maxwell and Cole, 2007). However, a previous prospective study provided evidence consistent with temporal ordering among these constructs (Liu et al., 2024). In sum, our indirect association analyses reflect between-subject correlations measured at one time point rather than naturalistic or experimentally induced changes.

Another important limitation is our self-reported physical activity measure. Our sample reported a high total amount of physical activity, consistent with evidence that self-report is susceptible to recall error and over-reporting, particularly for vigorous and moderate activity (Lee et al., 2011). In addition, participants without a valid IPAQ summary outcome had lower functional health literacy scores than those with valid outcomes. Although full-information maximum likelihood and complete-case analyses produced the same substantive indirect-association pattern, this descriptive difference indicates that IPAQ outcome availability was not homogeneous across all measured characteristics.

Measurement differences limited comparisons across the health literacy instruments. Because their response formats, content, breadth, and score distributions varied simultaneously, we could not determine which features contributed to the different patterns of association. Shared method variance may also have strengthened associations among the self-reported measures.

While we found no substantive differences in our findings after adjusting for whether participants submitted their data in person or online, we did not randomly assign the collection mode. Thus, our mode-adjusted sensitivity analyses cannot distinguish effects of the administration context from preexisting differences associated with participant recruitment or self-selection.

Age and sex were the only pre-specified covariates included across the principal analyses. Not comprehensively measuring previous exercise participation, health limitations, socioeconomic circumstances, and other behavioral or environmental determinants leaves the possibility of residual confounding. In addition, diagnostics indicated heteroscedasticity in the general health literacy regressions and the potential for functional-form mis-specification in the extended raw-total-activity models. The log-transformed outcome sensitivity analysis retained the substantive pattern, but these diagnostic findings reinforce the need for caution in interpreting the linear model estimates.

Finally, while our repeated cross-validation scheme provided an estimate of internal validation, the question of ecological validity remains. Similarly, our sample was young, mostly female, and drawn from a single Midwestern university. Moreover, even the self-efficacy models for vigorous activity showed modest performance, limiting potential of our models for individual-level prediction.

### 4.5 Implications for practice and future research

The present cross-sectional findings provide a rationale for longitudinal and experimental tests of whether combining health literacy content with self-efficacy strategies improves subsequent physical activity more than either approach alone. However, our findings do not show that a combined intervention would be superior (Bi et al., 2024). Candidate self-efficacy strategies for such trials include graded goals, mastery experiences, action planning, tailored feedback, and barrier management (Bandura, 1977; McAnally and Hagger, 2023; Maselli et al., 2018; Tang et al., 2019; Zhou et al., 2016). The exploratory activity-specific results provide a rationale for estimating intervention effects separately for vigorous activity, moderate activity, and walking. Trials could also test whether components addressing environmental access and opportunities embedded in daily routines improve walking (Rebar et al., 2016; Horacek et al., 2018). Such studies should assess whether combined interventions change exercise self-efficacy, whether those changes precede changes in physical activity, and whether effects differ across device-measured vigorous activity, moderate activity, and walking.

Broader studies could also incorporate social support, behavioral intention, opportunity, and environmental constraints to determine whether exercise self-efficacy contributes incrementally after accounting for additional social-cognitive and environmental determinants (Brown et al., 2024; Liu et al., 2024). Recent research linking digital health literacy with self-efficacy among college students provides further rationale for testing combined approaches (Zhou et al., 2025). Replication and external validation in more diverse universities and non-student populations will help to determine whether the observed patterns generalize beyond the population examined here.

## 5 Conclusion

Across models using three health literacy measures, adding exercise self-efficacy improved explained variance and held-out prediction beyond health literacy alone, whereas adding health literacy to models that already included self-efficacy did not improve held-out prediction. Absolute predictive performance nevertheless remained modest. In exploratory component analyses, adding self-efficacy produced larger predictive gains for vigorous and moderate activity than for walking, although we did not directly contrast the components. Our findings support cross-sectional indirect associations involving exercise self-efficacy for perceived digital and general health literacy, but not for performance-based functional health literacy. For the perceived measures, component-specific indirect associations met the false-discovery-rate-adjusted threshold for vigorous and moderate activity but not for walking. Overall, these results support considering exercise self-efficacy as a theoretically relevant construct in health literacy-centered models of physical activity. Future longitudinal and intervention studies should test the proposed temporal ordering and causal processes and directly compare associations across activity intensities and closely aligned perceived and performance-based measures of health literacy.

## Data Availability

The datasets presented in this article are not readily available because of restrictions in the participant consent materials. Analysis code and aggregate statistical results that do not disclose participant-level records are publicly available on the Open Science Framework at https://osf.io/wjyq8/. Requests to access the datasets should be directed to Ben Allen; benallen@ku.edu.

## References

[ref1] Allen M. S. Vella S. A. (2015). Longitudinal determinants of walking, moderate, and vigorous physical activity in Australian adults. Prev. Med. 78, 101–104. doi: 10.1016/j.ypmed.2015.07.014, 26212630

[ref2] Arlinghaus K. R. Johnston C. A. (2017). Advocating for behavior change with education. Am. J. Lifestyle Med. 12, 113–116. doi: 10.1177/1559827617745479, 30283247 PMC6124997

[ref3] Bandura A. (1977). Self-efficacy: toward a unifying theory of behavioral change. Psychol. Rev. 84, 191–215. doi: 10.1037/0033-295x.84.2.191, 847061

[ref4] Benjamini Y. Hochberg Y. (1995). Controlling the false discovery rate: a practical and powerful approach to multiple testing. J. R. Stat. Soc. Ser. B Stat. Methodol. 57, 289–300. doi: 10.1111/j.2517-6161.1995.tb02031.x

[ref5] Bi S. Yuan J. Wang Y. Zhang W. Zhang L. Zhang Y. . (2024). Effectiveness of digital health interventions in promoting physical activity among college students: systematic review and meta-analysis. J. Med. Internet Res. 26:e51714. doi: 10.2196/51714, 39566049 PMC11618011

[ref6] Brown C. E. B. Richardson K. Halil-Pizzirani B. Atkins L. Yücel M. Segrave R. A. (2024). Key influences on university students’ physical activity: a systematic review using the theoretical domains framework and the COM-B model of human behaviour. BMC Public Health 24:418. doi: 10.1186/s12889-023-17621-4, 38336748 PMC10854129

[ref7] Buja A. Rabensteiner A. Sperotto M. Grotto G. Bertoncello C. Cocchio S. . (2020). Health literacy and physical activity: a systematic review. J. Phys. Act. Health 17, 1259–1274. doi: 10.1123/jpah.2020-0161 33129198

[ref8] Craig C. L. Marshall A. L. Sjöström M. Bauman A. E. Booth M. L. Ainsworth B. E. . (2003). International physical activity questionnaire: 12-country reliability and validity. Med. Sci. Sports Exerc. 35, 1381–1395. doi: 10.1249/01.MSS.0000078924.61453.FB 12900694

[ref9] Edwards G. Dorstyn D. Oxlad M. (2025). Examining the relationship between health literacy and eHealth literacy in adult populations: a systematic review and meta-analysis. Health Promot. Int. 40:daaf217. doi: 10.1093/heapro/daaf217, 41398315 PMC12705269

[ref10] Geboers B. de Winter A. F. Luten K. A. Jansen C. J. M. Reijneveld S. A. (2014). The association of health literacy with physical activity and nutritional behavior in older adults, and its social cognitive mediators. J. Health Commun. 19, 61–76. doi: 10.1080/10810730.2014.934933, 25315584

[ref11] Ginis K. A. M. Nigg C. R. Smith A. L. (2013). Peer-delivered physical activity interventions: an overlooked opportunity for physical activity promotion. Transl. Behav. Med. 3, 434–443. doi: 10.1007/s13142-013-0215-2, 24294332 PMC3830020

[ref12] Hayes A. F. (2022). Introduction to Mediation, Moderation, and Conditional Process Analysis: A Regression-Based Approach. 3rd Edn. New York, NY, United States: Guilford Press.

[ref13] Hood K. K. Hilliard M. Piatt G. Ievers-Landis C. E. (2015). Effective strategies for encouraging behavior change in people with diabetes. Diabetes Manag. 5, 499–510. doi: 10.2217/dmt.15.43 PMC6086609 30100925

[ref14] Horacek T. M. Dede Yildirim E. Kattelmann K. Brown O. Byrd-Bredbenner C. Colby S. . (2018). Path analysis of campus walkability/bikeability and college students’ physical activity attitudes, behaviors, and body mass index. Am. J. Health Promot. 32, 578–586. doi: 10.1177/0890117116666357, 27630109

[ref15] Huang K. Beckman E. M. Ng N. Dingle G. A. Han R. James K. . (2024). Effectiveness of physical activity interventions on undergraduate students’ mental health: systematic review and meta-analysis. Health Promot. Int. 39:daae054. doi: 10.1093/heapro/daae054, 38916148 PMC11196957

[ref16] Kljajević V. Stanković M. Đorđević D. Trkulja-Petković D. Jovanović R. Plazibat K. . (2021). Physical activity and physical fitness among university students: a systematic review. Int. J. Environ. Res. Public Health 19:158. doi: 10.3390/ijerph19010158, 35010418 PMC8750240

[ref17] Lawrence A. Wardle J. Yoxall J. (2026). The relationship between health literacy and adherence to physical activity guidelines in adults: a scoping review. Am. J. Health Promot. 40, 378–391. doi: 10.1177/08901171251377053, 40913784 PMC12861545

[ref18] Ledermann T. Rudaz M. Fritz M. S. (2025). On partial versus full mediation and the importance of effect sizes. Adv. Methods Pract. Psychol. Sci. 8:25152459251355585. doi: 10.1177/25152459251355585

[ref19] Lee P. H. Macfarlane D. J. Lam T. H. Stewart S. M. (2011). Validity of the international physical activity questionnaire short form (IPAQ-SF): a systematic review. Int. J. Behav. Nutr. Phys. Act. 8:115. doi: 10.1186/1479-5868-8-115, 22018588 PMC3214824

[ref20] Lee I. M. Shiroma E. J. Lobelo F. Puska P. Blair S. N. Katzmarzyk P. T. (2012). Effect of physical inactivity on major non-communicable diseases worldwide: an analysis of burden of disease and life expectancy. Lancet 380, 219–229. doi: 10.1016/S0140-6736(12)61031-9, 22818936 PMC3645500

[ref21] Liu H. X. Chow B. C. Hassel H. Huang Y. W. Liang W. Wang R. B. (2024). Prospective association of eHealth literacy and health literacy with physical activity among Chinese college students: a multiple mediation analysis. Front. Public Health 12:1275691. doi: 10.3389/fpubh.2024.1275691, 38389943 PMC10881736

[ref22] Florence Health Literacy Research Group Lorini C. Lastrucci V. Mantwill S. Vettori V. Bonaccorsi G. (2019). Measuring health literacy in Italy: a validation study of the HLS-EU-Q16 and of the HLS-EU-Q6 in Italian language, conducted in Florence and its surroundings. Ann. Ist. Super. Sanita 55, 10–18. doi: 10.4415/ANN_19_01_04 30968831

[ref23] Maselli M. Ward P. B. Gobbi E. Carraro A. (2018). Promoting physical activity among university students: a systematic review of controlled trials. Am. J. Health Promot. 32, 1602–1612. doi: 10.1177/0890117117753798, 29366334

[ref24] Matthews J. A. Matthews S. Faries M. D. Wolever R. Q. (2024). Supporting sustainable health behavior change: the whole is greater than the sum of its parts. Mayo Clin. Proc. Innov. Qual. Outcomes 8, 263–275. doi: 10.1016/j.mayocpiqo.2023.10.002, 38807973 PMC11130595

[ref25] Maxwell S. E. Cole D. A. (2007). Bias in cross-sectional analyses of longitudinal mediation. Psychol. Methods 12, 23–44. doi: 10.1037/1082-989X.12.1.23, 17402810

[ref26] McAnally K. Hagger M. S. (2023). Health literacy, social cognition constructs, and health behaviors and outcomes: a meta-analysis. Health Psychol. 42, 213–234. doi: 10.1037/hea0001266, 37023324

[ref27] Moriishi C. Takano K. Oba T. Konishi N. Katahira K. Kimura K. (2026). Examining the factor structure of objective health literacy and numeracy scales: large-scale cross-sectional study. JMIR Public Health Surveill. 12:e71701. doi: 10.2196/71701, 41494165 PMC12779097

[ref28] Norman C. D. Skinner H. A. (2006). eHEALS: the eHealth literacy scale. J. Med. Internet Res. 8:e27. doi: 10.2196/jmir.8.4.e27, 17213046 PMC1794004

[ref29] Nutbeam D. (2008). The evolving concept of health literacy. Soc. Sci. Med. 67, 2072–2078. doi: 10.1016/j.socscimed.2008.09.050, 18952344

[ref30] Pelikan J. M. Ganahl K. (2017). Measuring health literacy in general populations: primary findings from the HLS-EU consortium’s health literacy assessment effort. Stud. Health Technol. Inform. 240, 34–59. 28972508

[ref31] Rebar A. L. Maher J. P. Doerksen S. E. Elavsky S. Conroy D. E. (2016). Intention-behavior gap is wider for walking and moderate physical activity than for vigorous physical activity in university students. J. Sci. Med. Sport 19, 130–134. doi: 10.1016/j.jsams.2014.11.392, 25529743

[ref32] Resnick B. Jenkins L. S. (2000). Testing the reliability and validity of the self-efficacy for exercise scale. Nurs. Res. 49, 154–159. doi: 10.1097/00006199-200005000-00007, 10882320

[ref33] Rovniak L. S. Anderson E. S. Winett R. A. Stephens R. S. (2002). Social cognitive determinants of physical activity in young adults: a prospective structural equation analysis. Ann. Behav. Med. 24, 149–156. doi: 10.1207/S15324796ABM2402_12, 12054320

[ref34] Sørensen K. Van den Broucke S. Fullam J. Doyle G. Pelikan J. Slonska Z. . (2012). Health literacy and public health: a systematic review and integration of definitions and models. BMC Public Health 12:80. doi: 10.1186/1471-2458-12-80, 22276600 PMC3292515

[ref35] Tang M. Y. Smith D. M. Mc Sharry J. Hann M. French D. P. (2019). Behavior change techniques associated with changes in postintervention and maintained changes in self-efficacy for physical activity: a systematic review with meta-analysis. Ann. Behav. Med. 53, 801–815. doi: 10.1093/abm/kay090, 30534971

[ref36] Weiss B. D. Mays M. Z. Martz W. Castro K. M. DeWalt D. A. Pignone M. P. . (2005). Quick assessment of literacy in primary care: the newest vital sign. Ann. Fam. Med. 3, 514–522. doi: 10.1370/afm.405 PMC1466931 16338915

[ref37] Wendel-Vos W. Droomers M. Kremers S. Brug J. Van Lenthe F. (2007). Potential environmental determinants of physical activity in adults: a systematic review. Obes. Rev. 8, 425–440. doi: 10.1111/j.1467-789X.2007.00370.x, 17716300

[ref38] Young M. D. Plotnikoff R. C. Collins C. E. Callister R. Morgan P. J. (2014). Social cognitive theory and physical activity: a systematic review and meta-analysis. Obes. Rev. 15, 983–995. doi: 10.1111/obr.12225, 25428600

[ref39] Zhou G. Wang D. Knoll N. Schwarzer R. (2016). Planning mediates between self-efficacy and physical activity among motivated young adults. J. Phys. Act. Health 13, 87–93. doi: 10.1123/jpah.2014-0555, 25830660

[ref40] Zhou Y. Xu J. Wang R. Guan X. (2025). Understanding how digital health literacy affects health self-management behaviors: the mediating role of self-efficacy in college students. Sci. Rep. 15:27230. doi: 10.1038/s41598-025-12726-9, 40715348 PMC12297381

