# Supplementary material for: Health literacy, exercise self-efficacy, and physical activity in university students: a cross-sectional study
Source: Front Psychol. 2026 Oct 30;17:1863857. doi: 10.3389/fpsyg.2026.1863857 (PMC13760528; doi:10.3389/fpsyg.2026.1863857)
Supplement: Supplementary file 1 [file Supplementary_file_1.docx]

**Supplementary Material**

Health literacy, exercise self-efficacy, and physical activity in university students: a cross-sectional study

Ben Allen

# **Table S1. Missingness in the analytic base sample**

| **Variable** | **Missing n** | **Missing proportion** |
| --- | --- | --- |
| eHEALS total | 0 | 0.000 |
| General HL index | 16 | 0.027 |
| Newest Vital Sign | 0 | 0.000 |
| Exercise self-efficacy total | 1 | 0.002 |
| IPAQ total MET-min/week | 32 | 0.054 |
| Age | 3 | 0.005 |
| Female | 3 | 0.005 |
| IPAQ vigorous MET-min/week | 32 | 0.054 |
| IPAQ moderate MET-min/week | 32 | 0.054 |
| IPAQ walking MET-min/week | 32 | 0.054 |
| IPAQ sitting min/weekday | 9 | 0.015 |
| IPAQ activity category | 32 | 0.054 |

Note. Analytic base N = 592. IPAQ processing resulted in 32/592 (5.4%) missing vigorous, moderate, walking, total, and category outcomes; valid IPAQ summary n = 560.

# **Table S2. Pairwise Pearson correlations among primary variables**

| **Variable** | **eHEALS** | **GHL** | **NVS** | **SEE** | **IPAQ total** | **Age** | **Female** |
| --- | --- | --- | --- | --- | --- | --- | --- |
| eHEALS | 1.000 | 0.409 | 0.046 | 0.152 | 0.108 | -0.025 | -0.041 |
| GHL | 0.409 | 1.000 | 0.121 | 0.157 | 0.125 | -0.087 | 0.132 |
| NVS | 0.046 | 0.121 | 1.000 | 0.002 | -0.049 | -0.000 | -0.001 |
| SEE | 0.152 | 0.157 | 0.002 | 1.000 | 0.387 | -0.069 | -0.227 |
| IPAQ total | 0.108 | 0.125 | -0.049 | 0.387 | 1.000 | -0.064 | -0.124 |
| Age | -0.025 | -0.087 | -0.000 | -0.069 | -0.064 | 1.000 | -0.189 |
| Female | -0.041 | 0.132 | -0.001 | -0.227 | -0.124 | -0.189 | 1.000 |

*Note. Correlations use pairwise complete observations. Female is coded 1 = female, 0 = male.*

# **Table S3. HLS-EU-Q16 scoring audit**

| **Item response range** | **Minimum answered items** | **Index formula** | **Theoretical range** | **Scored n** | **Missing n** | **Observed minimum** | **Observed median** | **Observed maximum** | **Verification** |
| --- | --- | --- | --- | --- | --- | --- | --- | --- | --- |
| 1-4 | 14 | (mean item response - 1) * (50/3) | 0-50 | 576 | 16 | 12.500 | 34.375 | 50.000 | PASS |

# **Table S4. IPAQ activity-category reconciliation**

| **Category** | **Incoming derived n** | **Recomputed n** |
| --- | --- | --- |
| Low | 23 | 23 |
| Moderate | 149 | 149 |
| High | 388 | 388 |
| Missing | 32 | 32 |

Note. Thirty-two analytic-base participants have no IPAQ category because they were excluded during processing (12 >960 min/day; 20 positive activity days with missing duration).

# **Table S5. Detailed adjusted global model results**

| **Model** | **Regression sample, n** | **SEM fitted n** | **a β** | **b β** | **c′ β** | **Indirect Association** | | | **Total Association** | | | | **Full R²** | **ΔR²** | **ΔR²**  **CI low** | **ΔR²  CI high** |
| --- | --- | --- | --- | --- | --- | --- | --- | --- | --- | --- | --- | --- | --- | --- | --- | --- |
|  |  |  |  |  |  | **β** | **CI low** | **CI high** | **B** | **CI low** | **CI high** | **β** |  |  |  |  |
| Digital Health Literacy | 554 | 592 | 0.138 | 0.367 | 0.049 | 0.051 | 0.017 | 0.090 | 55.272 | 4.391 | 104.019 | 0.099 | 0.154 | 0.120 | 0.066 | 0.186 |
| General Health Literacy | 541 | 592 | 0.185 | 0.360 | 0.064 | 0.067 | 0.031 | 0.107 | 59.192 | 11.859 | 107.065 | 0.130 | 0.159 | 0.112 | 0.058 | 0.178 |
| Functional Health Literacy | 554 | 592 | 0.005 | 0.374 | -0.053 | 0.002 | -0.029 | 0.032 | -89.020 | -239.508 | 60.337 | -0.052 | 0.155 | 0.128 | 0.069 | 0.193 |

*Note. SEM estimates use FIML; regression R² and ΔR² use complete cases. B denotes an unstandardized coefficient, and β denotes a standardized coefficient. Bootstrap procedures requested 5,000 resamples.*

# **Table S6. Repeated 10-fold cross-validation for global models**

| **Model** | **Pooled OOF repeats** | **Health-literacy- centered R², mean (SD)** | **Self-efficacy- extended R², mean (SD)** | **ΔR², mean (SD)** | **Health literacy- centered RMSE** | **Self-efficacy- extended RMSE** | **OOF n/repeat** | **Folds** | **Imputations/fold** |
| --- | --- | --- | --- | --- | --- | --- | --- | --- | --- |
| Digital Health Literacy | 5 | 0.012 (0.004) | 0.134 (0.003) | 0.122 (0.004) | 3162.4 | 2961.1 | 560 | 10 | 5 |
| General Health Literacy | 5 | 0.022 (0.003) | 0.133 (0.003) | 0.110 (0.002) | 3145.7 | 2963.2 | 560 | 10 | 5 |
| Functional Health Literacy | 5 | 0.007 (0.003) | 0.134 (0.003) | 0.127 (0.006) | 3170.5 | 2960.8 | 560 | 10 | 5 |

Note. OOF = out of fold. Predictive R² = 1 − SSE/SST on held-out observations. Within each of five repeats, predictions from the 10 held-out folds were pooled before R² and RMSE were calculated; the table reports the arithmetic mean across the five repeat-specific pooled estimates. Health-literacy-centered and self-efficacy-extended models used the same imputed training data within each fold, and held-out outcomes were neither imputed nor used as imputation predictors.

# **Table S7. Component indirect associations and cross-validated prediction**

| **Model** | **Component** | **Indirect β** | **CI low** | **CI high** | **BH q** | **FDR supported** | **In-sample ΔR²** | **ΔR²  CI low** | **ΔR²  CI high** | **Health literacy- centered predictive R²** | **Self- Efficacy- extended predictive R²** | **Predictive ΔR²** |
| --- | --- | --- | --- | --- | --- | --- | --- | --- | --- | --- | --- | --- |
| Digital Health Literacy | vigorous | 0.0568 | 0.0209 | 0.0932 | 0.0035 | Yes | 0.1519 | 0.1005 | 0.2063 | 0.037 | 0.193 | 0.156 |
| Digital Health Literacy | moderate | 0.0298 | 0.0099 | 0.0556 | 0.0167 | Yes | 0.0401 | 0.0126 | 0.0794 | -0.000 | 0.039 | 0.039 |
| Digital Health Literacy | walking | 0.0147 | 0.0020 | 0.0322 | 0.1043 | No | 0.0130 | 0.0013 | 0.0331 | 0.010 | 0.019 | 0.009 |
| General Health Literacy | vigorous | 0.0770 | 0.0354 | 0.1205 | 0.0011 | Yes | 0.1457 | 0.0953 | 0.2012 | 0.043 | 0.196 | 0.153 |
| General Health Literacy | moderate | 0.0426 | 0.0176 | 0.0738 | 0.0104 | Yes | 0.0469 | 0.0160 | 0.0916 | -0.004 | 0.041 | 0.045 |
| General Health Literacy | walking | 0.0192 | 0.0020 | 0.0415 | 0.1043 | No | 0.0132 | 0.0012 | 0.0334 | 0.017 | 0.024 | 0.008 |
| Functional Health Literacy | vigorous | 0.0024 | -0.0328 | 0.0370 | 0.8930 | No | 0.1607 | 0.1083 | 0.2185 | 0.028 | 0.191 | 0.163 |
| Functional Health Literacy | moderate | 0.0010 | -0.0167 | 0.0201 | 0.9092 | No | 0.0428 | 0.0146 | 0.0843 | 0.001 | 0.041 | 0.040 |
| Functional Health Literacy | walking | 0.0006 | -0.0082 | 0.0105 | 0.9025 | No | 0.0121 | 0.0012 | 0.0325 | 0.012 | 0.019 | 0.007 |

Note. Component outcomes were modeled as log(1 + MET-min/week). β denotes a standardized coefficient. BH q-values were calculated separately within vigorous, moderate, and walking families. Predictive R² values are arithmetic means of five repeat-specific pooled out-of-fold estimates; within each repeat, predictions from all 10 held-out folds were pooled before R² was calculated.

# **Table S8. Sensitivity analysis using log(1 + total MET-min/week)**

| **Health literacy model** | **Indirect β** | **CI low** | **CI high** | **In-sample ΔR²** | **ΔR² CI low** | **ΔR² CI high** | **Health literacy- centered  predictive R²,  mean (SD)** | **Self-efficacy- extended predictive R²,  mean (SD)** | **Predictive ΔR², mean (SD)** |
| --- | --- | --- | --- | --- | --- | --- | --- | --- | --- |
| Digital Health Literacy | 0.053 | 0.019 | 0.090 | 0.127 | 0.071 | 0.198 | −0.010 (0.030) | 0.116 (0.027) | 0.126 (0.004) |
| General Health Literacy | 0.075 | 0.034 | 0.126 | 0.143 | 0.088 | 0.202 | −0.011 (0.004) | 0.104 (0.002) | 0.116 (0.004) |
| Functional Health Literacy | 0.006 | −0.027 | 0.042 | 0.133 | 0.076 | 0.207 | −0.016 (0.024) | 0.109 (0.019) | 0.126 (0.009) |

*Note. Indirect associations are standardized (β). In-sample ΔR² values are from complete-case regressions adjusted for age and sex. Predictive R² values are means (SDs) of five repeat-specific pooled out-of-fold estimates. Within each repeat, predictions from all 10 held-out folds were pooled before R² was calculated; five imputations were fitted within each training fold. The health literacy-centered model included health literacy, age, and sex; the self-efficacy-extended model additionally included exercise self-efficacy. All models used log(1 + total MET-min/week) as the outcome. Negative predictive R² indicates worse prediction than the held-out mean benchmark.*

# **Table S9. Adjusted-versus-unadjusted global comparison**

| **Health-literacy model** | **Covariate specification** | **a path,  B [95% CI]; β** | **b path,  B [95% CI]; β** | **c′ path,  B [95% CI]; β** | **Indirect association,  β [95% CI]** | **Total association, B [95% CI]; β** | **In-sample ΔR² [95% CI]** | **Full-model R² [95% CI]** |
| --- | --- | --- | --- | --- | --- | --- | --- | --- |
| Digital Health Literacy | Adjusted | 0.482  [0.174, 0.785];  β = 0.138 | 58.462  [43.366, 73.318];  β = 0.367 | 27.066  [-21.164, 74.887];  β = 0.049 | 0.051  [0.017, 0.090] | 55.272  [4.391, 104.019];  β = 0.099 | 0.120  [0.066, 0.186] | 0.154  [0.102, 0.229] |
| Digital Health Literacy | Unadjusted | 0.528  [0.226, 0.827];  β = 0.151 | 60.532  [46.420, 75.129];  β = 0.379 | 28.113  [-19.284, 74.812];  β = 0.050 | 0.057  [0.023, 0.095] | 60.083  [11.374, 108.943];  β = 0.108 | 0.141  [0.081, 0.207] | 0.153  [0.095, 0.220] |
| General Health Literacy | Adjusted | 0.527  [0.266, 0.773];  β = 0.185 | 57.361  [41.822, 72.300];  β = 0.360 | 28.989  [-19.384, 77.985];  β = 0.064 | 0.067  [0.031, 0.107] | 59.192  [11.859, 107.065];  β = 0.130 | 0.112  [0.058, 0.178] | 0.159  [0.103, 0.234] |
| General Health Literacy | Unadjusted | 0.448  [0.176, 0.709];  β = 0.157 | 60.166  [45.440, 74.347];  β = 0.377 | 25.519  [-20.762, 73.182];  β = 0.056 | 0.059  [0.022, 0.100] | 52.444  [4.787, 101.005];  β = 0.116 | 0.141  [0.080, 0.211] | 0.157  [0.098, 0.231] |
| Functional Health Literacy | Adjusted | 0.054  [-0.840, 0.918];  β = 0.005 | 59.658  [45.034, 74.041];  β = 0.374 | -92.227  [-237.538, 49.498];  β = -0.053 | 0.002  [-0.029, 0.032] | -89.020  [-239.508, 60.337];  β = -0.052 | 0.128  [0.069, 0.193] | 0.155  [0.099, 0.226] |
| Functional Health Literacy | Unadjusted | 0.048  [-0.868, 0.987];  β = 0.004 | 61.855  [47.586, 75.802];  β = 0.388 | -92.010  [-241.127, 46.461];  β = -0.053 | 0.002  [-0.031, 0.035] | -89.062  [-252.180, 59.840]; β = -0.052 | 0.150  [0.089, 0.219] | 0.153  [0.095, 0.223] |

*Note. Adjusted models include age and sex; unadjusted models omit these covariates. The a path links health literacy with exercise self-efficacy; the b path links exercise self-efficacy with total activity conditional on health literacy; c′ is the direct association. B denotes an unstandardized coefficient, and β denotes a standardized coefficient. Indirect associations are standardized. Bracketed ranges are 95% bootstrap confidence intervals. R² and ΔR² are complete-case regression statistics.*

# **Table S10. Adjusted-versus-unadjusted component comparison**

| **Health literacy model** | **Physical activity component** | **Covariate specification** | **Indirect association,  β [95% CI]** | **FDR-supported** | **In-sample ΔR² [95% CI]** | **Total association, B [95% CI]** |
| --- | --- | --- | --- | --- | --- | --- |
| Digital Health Literacy | Vigorous activity | Adjusted | 0.057 [0.021, 0.093] | Yes | 0.152 [0.100, 0.206] | 0.056 [0.008, 0.105] |
| Digital Health Literacy | Vigorous activity | Unadjusted | 0.066 [0.027, 0.107] | Yes | 0.188 [0.133, 0.248] | 0.063 [0.015, 0.111] |
| Digital Health Literacy | Moderate activity | Adjusted | 0.030 [0.010, 0.056] | Yes | 0.040 [0.013, 0.079] | 0.026 [-0.011, 0.063] |
| Digital Health Literacy | Moderate activity | Unadjusted | 0.035 [0.012, 0.063] | Yes | 0.053 [0.020, 0.099] | 0.030 [-0.006, 0.066] |
| Digital Health Literacy | Walking | Adjusted | 0.015 [0.002, 0.032] | No | 0.013 [0.001, 0.033] | -0.007 [-0.022, 0.008] |
| Digital Health Literacy | Walking | Unadjusted | 0.015 [0.000, 0.037] | No | 0.010 [0.000, 0.035] | -0.006 [-0.022, 0.009] |
| General Health Literacy | Vigorous activity | Adjusted | 0.077 [0.035, 0.120] | Yes | 0.146 [0.095, 0.201] | 0.042 [0.002, 0.082] |
| General Health Literacy | Vigorous activity | Unadjusted | 0.070 [0.029, 0.116] | Yes | 0.192 [0.136, 0.252] | 0.030 [-0.010, 0.070] |
| General Health Literacy | Moderate activity | Adjusted | 0.043 [0.018, 0.074] | Yes | 0.047 [0.016, 0.092] | 0.001 [-0.031, 0.031] |
| General Health Literacy | Moderate activity | Unadjusted | 0.039 [0.014, 0.071] | Yes | 0.065 [0.028, 0.113] | -0.004 [-0.036, 0.029] |
| General Health Literacy | Walking | Adjusted | 0.019 [0.002, 0.042] | No | 0.013 [0.001, 0.033] | 0.002 [-0.012, 0.015] |
| General Health Literacy | Walking | Unadjusted | 0.014 [-0.001, 0.037] | No | 0.008 [0.000, 0.032] | 0.007 [-0.007, 0.023] |
| Functional Health Literacy | Vigorous activity | Adjusted | 0.002 [-0.033, 0.037] | No | 0.161 [0.108, 0.219] | -0.026 [-0.161, 0.119] |
| Functional Health Literacy | Vigorous activity | Unadjusted | 0.002 [-0.035, 0.040] | No | 0.199 [0.142, 0.263] | -0.025 [-0.166, 0.119] |
| Functional Health Literacy | Moderate activity | Adjusted | 0.001 [-0.017, 0.020] | No | 0.043 [0.015, 0.084] | -0.048 [-0.162, 0.069] |
| Functional Health Literacy | Moderate activity | Unadjusted | 0.001 [-0.020, 0.022] | No | 0.057 [0.023, 0.104] | -0.048 [-0.164, 0.072] |
| Functional Health Literacy | Walking | Adjusted | 0.001 [-0.008, 0.011] | No | 0.012 [0.001, 0.032] | 0.006 [-0.054, 0.071] |
| Functional Health Literacy | Walking | Unadjusted | 0.000 [-0.008, 0.011] | No | 0.009 [0.000, 0.036] | 0.005 [-0.054, 0.073] |

*Note. Adjusted models include age and sex; unadjusted models omit these covariates. Component outcomes were modeled as log(1 + MET-min/week). B denotes an unstandardized coefficient on the log-transformed outcome scale, and β denotes a standardized coefficient. Indirect-association intervals are 95% percentile-bootstrap confidence intervals. FDR-supported indicates qBH < .05 after Benjamini–Hochberg adjustment within the relevant component family (digital-, general-, and functional-health-literacy indirect hypotheses; m = 3). The bootstrap intervals and FDR decisions are distinct inferential summaries; see Table S19 for the unadjusted p-values and adjusted q-values.*

# **Table S11. Bootstrap-resampling audit**

| **Analysis specification** | **Health literacy model** | **Physical activity outcome** | **Requested draws** | **Valid draws** | **Failed/ nonconvergent draws** | **Valid proportion** | **Draws extracted by manymome** |
| --- | --- | --- | --- | --- | --- | --- | --- |
| Primary adjusted total-activity model | Digital Health Literacy | Total activity | 5000 | 4996 | 4 | 0.9992 | 4996 |
| Primary adjusted total-activity model | General Health Literacy | Total activity | 5000 | 4995 | 5 | 0.9990 | 4995 |
| Primary adjusted total-activity model | Functional Health Literacy | Total activity | 5000 | 4996 | 4 | 0.9992 | 4996 |
| Primary adjusted component model | Digital Health Literacy | Vigorous activity | 5000 | 4993 | 7 | 0.9986 | 4993 |
| Primary adjusted component model | Digital Health Literacy | Moderate activity | 5000 | 4979 | 21 | 0.9958 | 4979 |
| Primary adjusted component model | Digital Health Literacy | Walking | 5000 | 4995 | 5 | 0.9990 | 4995 |
| Primary adjusted component model | General Health Literacy | Vigorous activity | 5000 | 4992 | 8 | 0.9984 | 4992 |
| Primary adjusted component model | General Health Literacy | Moderate activity | 5000 | 4994 | 6 | 0.9988 | 4994 |
| Primary adjusted component model | General Health Literacy | Walking | 5000 | 5000 | 0 | 1.0000 | 5000 |
| Primary adjusted component model | Functional Health Literacy | Vigorous activity | 5000 | 4992 | 8 | 0.9984 | 4992 |
| Primary adjusted component model | Functional Health Literacy | Moderate activity | 5000 | 4997 | 3 | 0.9994 | 4997 |
| Primary adjusted component model | Functional Health Literacy | Walking | 5000 | 5000 | 0 | 1.0000 | 5000 |
| Log-transformed total-activity sensitivity | Digital Health Literacy | Total activity | 5000 | 4997 | 3 | 0.9994 | 4997 |
| Log-transformed total-activity sensitivity | General Health Literacy | Total activity | 5000 | 5000 | 0 | 1.0000 | 5000 |
| Log-transformed total-activity sensitivity | Functional Health Literacy | Total activity | 5000 | 4998 | 2 | 0.9996 | 4998 |
| Unadjusted total-activity model | Digital Health Literacy | Total activity | 5000 | 4994 | 6 | 0.9988 | 4994 |
| Unadjusted total-activity model | General Health Literacy | Total activity | 5000 | 4998 | 2 | 0.9996 | 4998 |
| Unadjusted total-activity model | Functional Health Literacy | Total activity | 5000 | 5000 | 0 | 1.0000 | 5000 |
| Unadjusted component model | Digital Health Literacy | Vigorous activity | 5000 | 4998 | 2 | 0.9996 | 4998 |
| Unadjusted component model | Digital Health Literacy | Moderate activity | 5000 | 5000 | 0 | 1.0000 | 5000 |
| Unadjusted component model | Digital Health Literacy | Walking | 5000 | 5000 | 0 | 1.0000 | 5000 |
| Unadjusted component model | General Health Literacy | Vigorous activity | 5000 | 4998 | 2 | 0.9996 | 4998 |
| Unadjusted component model | General Health Literacy | Moderate activity | 5000 | 5000 | 0 | 1.0000 | 5000 |
| Unadjusted component model | General Health Literacy | Walking | 5000 | 5000 | 0 | 1.0000 | 5000 |
| Unadjusted component model | Functional Health Literacy | Vigorous activity | 5000 | 5000 | 0 | 1.0000 | 5000 |
| Unadjusted component model | Functional Health Literacy | Moderate activity | 5000 | 4999 | 1 | 0.9998 | 4999 |
| Unadjusted component model | Functional Health Literacy | Walking | 5000 | 5000 | 0 | 1.0000 | 5000 |

Note. Each SEM requested 5,000 bootstrap attempts. manymome reused all valid bootstrap draws stored by lavaan; failed/nonconvergent refits were excluded. The primary global models retained 4,995 - 4,996 valid draws; the minimum across primary global and component models was 4,979/5,000 (99.58%).

# **Table S12. Analysis settings and software versions**

| **Setting** | **Value** |
| --- | --- |
| primary_outcome | ipaq_total_met_min_week |
| log1p_sensitivity_outcome | ipaq_total_met_min_week_log1p |
| regression_bootstrap_resamples | 5000 |
| sem_bootstrap_resamples | 5000 |
| bootstrap_ci_type | bootstrap_percentile |
| cv_folds | 10 |
| cv_repeats | 5 |
| cv_imputations | 5 |
| cv_r2_metric | yardstick::rsq_trad_vec; 1 - SSE/SST on held-out observations |
| component_outcomes_log1p | TRUE |
| hls_eu_q16_scoring | (mean of 1-4 items - 1) * (50/3), range 0-50 |
| hls_eu_q16_min_answered | 14 |
| sem_raw_total_scale_divisor | 1000 |
| minimum_valid_sem_bootstrap_fraction | 0.95 |
| expected_raw_dataset_n | 642 |
| expected_raw_dataset_p | 633 |
| expected_ipaq_valid_n | 560 |
| expected_ipaq_excluded_n | 32 |
| expected_ipaq_gt960_n | 12 |
| expected_ipaq_missing_duration_n | 20 |
| manymome_version | 0.2.9 |
| lavaan_version | 0.6.19 |
| yardstick_version | 1.3.1 |
| seed | 20260331 |

Note. The analysis retains the N = 592 analytic base while treating 32 protocol-invalid IPAQ activity summaries as missing outcomes.

# **Table S13. Internal consistency of multi-item measures in the analytic sample**

| **Scale** | **Items** | **Complete item sets, n** | **Cronbach's α** |
| --- | --- | --- | --- |
| Self-Efficacy for Exercise (SEE) | 9 | 588 | 0.928 |
| eHealth Literacy Scale (eHEALS) | 8 | 588 | 0.920 |
| HLS-EU-Q16 | 16 | 503 | 0.886 |
| Newest Vital Sign (NVS) | 6 | 592 | 0.750 |

Note. Cronbach's α was calculated using participants with complete item-level responses for each measure. Measure scores used in the main analyses followed the scoring and missing-item rules described in the manuscript and therefore can have larger analytic n than the complete-item reliability subset.

# **Table S14. Post hoc comparison of online and in-person data-collection modes**

| **Variable** | **Online** | **In person** | **Unadjusted p** |
| --- | --- | --- | --- |
| Age (years) | 346; 18.00 [18.00, 19.00] | 243; 18.00 [18.00, 19.00] | 0.753 |
| Female sex, n/N (%) | 245/345 (71.0%) | 172/244 (70.5%) | 0.964 |
| eHEALS total | 347; 32.00 [28.00, 33.00] | 245; 32.00 [28.00, 34.00] | 0.840 |
| General health literacy index | 337; 33.33 [30.21, 39.58] | 239; 35.42 [32.26, 40.62] | 0.048 |
| Newest Vital Sign | 347; 4.00 [2.00, 5.00] | 245; 5.00 [4.00, 6.00] | <0.001 |
| Exercise self-efficacy total | 346; 56.00 [42.25, 76.00] | 245; 56.00 [45.00, 70.00] | 0.496 |
| IPAQ total MET-min/week | 325; 4284.00 [2476.00, 6318.00] | 235; 3759.00 [2046.00, 5985.00] | 0.075 |

Note. Continuous variables are shown as n; median [IQR] and were compared with two-sided Mann-Whitney U tests. Sex is shown as n/N (%) and was compared with a chi-square test. These post hoc mode comparisons were descriptive and p values were not adjusted for multiplicity. Corrected total IPAQ activity was available for 325 online and 235 in-person participants. Questionnaire administration was electronic in both modes, but in-person participants also completed laboratory procedures.

# **Table S15. Collection-mode-adjusted sensitivity analyses for all three principal SEMs**

| **Health-literacy model** | **a path β** | **b path β** | **c′ path β** | **Indirect β [95% bootstrap CI]** | **Fitted nobs** |
| --- | --- | --- | --- | --- | --- |
| Digital Health Literacy | 0.138 | 0.365 | 0.048 | 0.050 [0.019, 0.089] | 592 |
| General Health Literacy | 0.188 | 0.356 | 0.072 | 0.067 [0.030, 0.109] | 592 |
| Functional Health Literacy | 0.012 | 0.372 | -0.040 | 0.004 [-0.028, 0.036] | 592 |

*Note.* All models included age, sex, and collection mode in both structural equations. Collection mode was coded 1 = online and 0 = in person. The models used FIML and requested 5,000 nonparametric bootstrap resamples.

# **Table S16. FIML implementation and fitted observations**

| **Health literacy model** | **Health-literacy measure (analysis variable)** | **Input rows** | **Complete rows** | **Fitted observations** | **lavaan settings** |
| --- | --- | --- | --- | --- | --- |
| Digital Health Literacy | eHEALS total (eheal_total) | 592 | 554 | 592 | fiml; fixed.x = FALSE;  meanstructure = TRUE |
| General Health Literacy | HLS-EU-Q16 index (ghl_index) | 592 | 541 | 592 | fiml; fixed.x = FALSE;  meanstructure = TRUE |
| Functional Health Literacy | Newest Vital Sign total (nvs_total) | 592 | 554 | 592 | fiml; fixed.x = FALSE;  meanstructure = TRUE |

# **Table S17. IPAQ summary-outcome availability**

**Panel A. Outcome-availability mechanisms**

| **IPAQ outcome status** | **Participants** | **Percent of N = 592** |
| --- | --- | --- |
| Unavailable: positive days with insufficient duration | 20 | 3.4% |
| Unavailable: pre-truncation total >960 min/day | 12 | 2.0% |
| Valid IPAQ summary outcome | 560 | 94.6% |

**Panel B. Descriptive comparison of participants with and without valid IPAQ outcomes**

| **Variable** | **Valid IPAQ outcome** | **Unavailable IPAQ outcome** | **SMD valid − unavailable** |
| --- | --- | --- | --- |
| Age, years | 18.66 (1.40);  18.00 [18.00, 19.00] (n = 558) | 18.77 (0.76);  19.00 [18.00, 19.00] (n = 31) | −0.08 |
| Female proportion | 70.6% (n = 557) | 75.0% (n = 32) | −0.10 |
| Exercise self-efficacy | 56.73 (19.98);  56.00 [43.00, 72.00] (n = 559) | 60.75 (19.26);  57.50 [48.75, 77.00] (n = 32) | −0.20 |
| Digital health literacy | 30.80 (5.73);  32.00 [28.00, 34.00] (n = 560) | 30.14 (5.34);  31.00 [27.86, 32.00] (n = 32) | 0.12 |
| General health literacy | 35.27 (6.93);  34.38 [31.25, 39.58] (n = 546) | 35.57 (8.40);  34.38 [31.51, 42.19] (n = 30) | −0.04 |
| Newest Vital Sign | 4.19 (1.79);  5.00 [3.00, 6.00] (n = 560) | 2.56 (2.17);  3.00 [0.00, 4.00] (n = 32) | 0.90 |
| Online collection proportion | 58.0% (n = 560) | 68.8% (n = 32) | −0.22 |

*Note.* Continuous variables are mean (SD); median [Q1, Q3]. Binary variables are percentages. SMD = standardized mean difference. These comparisons are descriptive; no independent-samples significance tests were used.

# **Table S18. Complete-case SEM sensitivity**

| **Health-literacy model** | **Primary FIML nobs** | **Primary indirect β [95% CI]** | **Complete-case n** | **Complete-case indirect β [95% CI]** | **Conclusion** |
| --- | --- | --- | --- | --- | --- |
| Digital Health Literacy | 592 | 0.051 [0.017, 0.090] | 554 | 0.048 [0.016, 0.085] | Supported |
| General Health Literacy | 592 | 0.067 [0.031, 0.107] | 541 | 0.082 [0.042, 0.126] | Supported |
| Functional Health Literacy | 592 | 0.002 [−0.029, 0.032] | 554 | 0.007 [−0.024, 0.038] | Not supported |

*Note.* Complete-case samples required observed health literacy, exercise self-efficacy, total MET-min/week, age, and sex. Both analyses used the same structural equations and requested 5,000 nonparametric bootstrap resamples.

# **Table S19. Reconstruction of component-specific Benjamini–Hochberg adjustments**

| **Multiplicity family** | **Health literacy hypothesis** | **Indirect β [95% percentile-bootstrap CI]** | **Unadjusted p** | **BH q** | **q < .05** |
| --- | --- | --- | --- | --- | --- |
| Vigorous | Digital Health Literacy | 0.057 [0.021, 0.093] | 0.0023 | 0.0035 | Yes |
| Vigorous | General Health Literacy | 0.077 [0.035, 0.120] | 0.0004 | 0.0011 | Yes |
| Vigorous | Functional Health Literacy | 0.002 [-0.033, 0.037] | 0.8930 | 0.8930 | No |
| Moderate | Digital Health Literacy | 0.030 [0.010, 0.056] | 0.0111 | 0.0167 | Yes |
| Moderate | General Health Literacy | 0.043 [0.018, 0.074] | 0.0035 | 0.0104 | Yes |
| Moderate | Functional Health Literacy | 0.001 [-0.017, 0.020] | 0.9092 | 0.9092 | No |
| Walking | Digital Health Literacy | 0.015 [0.002, 0.032] | 0.0696 | 0.1043 | No |
| Walking | General Health Literacy | 0.019 [0.002, 0.042] | 0.0693 | 0.1043 | No |
| Walking | Functional Health Literacy | 0.001 [-0.008, 0.011] | 0.9025 | 0.9025 | No |

*Note.* Vigorous, moderate, and walking were three separate multiplicity families; each family contained the digital-, general-, and functional-health-literacy indirect hypotheses (m = 3). The primary total-activity indirect hypotheses were evaluated separately with 95% percentile-bootstrap confidence intervals and were not included in the BH adjustments. Inputs to BH were lavaan's two-sided normal-reference p-values for the defined indirect parameter ab, calculated from the indirect estimate and its bootstrap standard error. The confidence intervals were calculated separately from percentiles of the empirical bootstrap distribution; therefore, exclusion of zero from a percentile-bootstrap interval is not mathematically equivalent to an unadjusted normal-reference p < .05.

# **Table S20. Newest Vital Sign score distribution**

| **NVS score** | **Participants** | **Percent of N = 592** |
| --- | --- | --- |
| 0 | 42 | 7.1% |
| 1 | 34 | 5.7% |
| 2 | 39 | 6.6% |
| 3 | 70 | 11.8% |
| 4 | 99 | 16.7% |
| 5 | 140 | 23.6% |
| 6 | 168 | 28.4% |

*Note.* A total of 168/592 participants (28.4%) achieved the maximum score of 6, and 308/592 (52.0%) scored either 5 or 6.

# **Table S21. HLS-EU-Q16 item completion and complete-item sensitivity analysis**

**Panel A. Number of answered HLS-EU-Q16 items**

| **Answered items** | **n** | **% of analytic base** | **% of scored sample** | **Scoring status** |
| --- | --- | --- | --- | --- |
| 0-13 (not scored) | 16 | 2.7% | — | Not scored |
| 14 | 22 | 3.7% | 3.8% | Scored |
| 15 | 51 | 8.6% | 8.9% | Scored |
| 16 | 503 | 85.0% | 87.3% | Scored |

**Panel B. Complete-16-item SEM sensitivity**

| **Analysis** | **Fitted nobs** | **Indirect β [95% bootstrap CI]** | **Substantive conclusion** |
| --- | --- | --- | --- |
| Primary score (≥14 answered items) | 592 | 0.067 [0.031, 0.107] | Supported |
| Complete 16-item responses only | 503 | 0.063 [0.026, 0.108] | Supported |

*Note.* The primary 0–50 index required at least 14 of 16 answered items. The sensitivity analysis retained only participants with all 16 items answered; other model specifications were unchanged.

# **Table S22. IPAQ hour-field correction audit and exclusion sensitivity**

**Panel A. Corrected hour-field entries**

| **Correction** | **Activity** | **Raw hours field** | **Raw minutes field** | **Scored duration (min/day)** | **Literal duration (min/day)** |
| --- | --- | --- | --- | --- | --- |
| 1 | Moderate | 60 | 0 | 60 | 3600 |
| 2 | Moderate | 30 | blank | 30 | 1800 |
| 3 | Walking | 30 | blank | 30 | 1800 |
| 4 | Moderate | 30 | blank | 30 | 1800 |
| 5 | Moderate | 30 | 0 | 30 | 1800 |
| 6 | Moderate | 45 | blank | 45 | 2700 |
| 7 | Walking | 15 | blank | 15 | 900 |
| 8 | Walking | 15 | blank | 15 | 900 |
| 9 | Vigorous | 15 | 0 | 15 | 900 |
| 10 | Walking | 30 | blank | 30 | 1800 |

**Panel B. Sensitivity analysis excluding all affected participant records**

| **Health-literacy model** | **Primary indirect  β [95% CI]** | **Exclusion nobs** | **Exclusion indirect  β [95% CI]** | **Interpretation** |
| --- | --- | --- | --- | --- |
| Digital Health Literacy | 0.051 [0.017, 0.090] | 583 | 0.051 [0.019, 0.089] | Unchanged |
| General Health Literacy | 0.067 [0.031, 0.107] | 583 | 0.067 [0.029, 0.109] | Unchanged |
| Functional Health Literacy | 0.002 [−0.029, 0.032] | 583 | 0.003 [-0.028, 0.033] | Unchanged |

*Note.* The 10 corrected fields occurred across nine participant records: eight participants had one correction and one had two. Corrected hour values were restricted to the specified minute-like set {15, 30, 45, 60, 90}; none had a positive accompanying minutes entry. Blank minutes were treated as zero. The same rule was applied without reference to outcome-model results. Panel B excludes all nine affected records.

# **Table S23. Paired-sample diagnostics for the primary nested regressions**

| **Health**  **Literacy model** | **Specification** | **n** | **Max leverage (flagged)** | **Max Cook's D (flagged)** | **Max \|studentized residual\| (flagged)** | **Koenker–BP χ² (df)** | **p** | **RESET F  (df1, df2)** | **p** |
| --- | --- | --- | --- | --- | --- | --- | --- | --- | --- |
| Digital Health Literacy | Health literacy-centered:  HL + age + sex | 554 | 0.349 (28) | 0.552 (26) | 3.984 (9) | 6.53 (3) | 0.088 | 2.19 (2, 548) | 0.113 |
| Digital Health Literacy | Self-efficacy- extended:  + exercise self-efficacy | 554 | 0.354 (26) | 0.464 (24) | 5.273 (7) | 7.64 (4) | 0.106 | 4.80 (2, 547) | 0.009 |
| General Health Literacy | Health literacy-centered:  HL + age + sex | 541 | 0.354 (23) | 0.506 (28) | 4.332 (7) | 10.59 (3) | 0.014 | 1.25 (2, 535) | 0.287 |
| General Health Literacy | Self-efficacy- extended:  + exercise self-efficacy | 541 | 0.358 (20) | 0.425 (25) | 5.220 (6) | 10.84 (4) | 0.028 | 5.46 (2, 534) | 0.005 |
| Functional Health Literacy | Health literacy-centered:  HL + age + sex | 554 | 0.349 (24) | 0.572 (25) | 4.139 (8) | 4.32 (3) | 0.229 | 2.90 (2, 548) | 0.056 |
| Functional Health Literacy | Self-efficacy- extended:  + exercise self-efficacy | 554 | 0.353 (26) | 0.471 (22) | 5.335 (7) | 7.45 (4) | 0.114 | 3.44 (2, 547) | 0.033 |

*Note.* Within each health-literacy analysis, the health-literacy-centered and self-efficacy-extended regressions were fit to the same complete-case sample. Parenthetical counts are observations above the prespecified screening thresholds: leverage >2p/n, Cook's D >4/n, and absolute externally studentized residual >3. Koenker–Breusch–Pagan tests indicated heteroscedasticity for both general-health-literacy specifications (p = .014 and .028). Ramsey RESET tests indicated possible functional-form departures for all three self-efficacy-extended models (p = .009, .005, and .033 for digital, general, and functional health literacy, respectively), but not for the health-literacy-centered models. These screens motivated retention of the log-transformed outcome sensitivity analysis; they were not used for data-driven case deletion.
